# The evolution of nanopore sequencing

**DOI:** 10.3389/fgene.2014.00449

**Published:** 2015-01-07

**Authors:** Yue Wang, Qiuping Yang, Zhimin Wang

**Affiliations:** Department of Plant Science, School of Agriculture and Biology, Shanghai Jiao Tong UniversityShanghai, China

**Keywords:** gold standards, third-generation sequencing, nanopore sequencing, ionic current blockage, DNA ratcheting, sequencing by tunneling, multiplexing detection, field-effect-transistor (FET) nanopore sensor

## Abstract

The “$1000 Genome” project has been drawing increasing attention since its launch a decade ago. Nanopore sequencing, the third-generation, is believed to be one of the most promising sequencing technologies to reach four gold standards set for the “$1000 Genome” while the second-generation sequencing technologies are bringing about a revolution in life sciences, particularly in genome sequencing-based personalized medicine. Both of protein and solid-state nanopores have been extensively investigated for a series of issues, from detection of ionic current blockage to field-effect-transistor (FET) sensors. A newly released protein nanopore sequencer has shown encouraging potential that nanopore sequencing will ultimately fulfill the gold standards. In this review, we address advances, challenges, and possible solutions of nanopore sequencing according to these standards.

## Introduction

DNA sequencing is the most powerful method to reveal genetic variations at the molecular level, such as single nucleotide polymorphism, copy number variation, gene fusion, and insertion/deletion, etc., which are relevant to genetic diseases including cancer (Topol, 2014). Hence, its importance in understanding of disease mechanism, genetic diagnosis, and personalized medicine (Hamburg and Collins, 2010) cannot be overestimated. In 2004, the National Human Genome Research Institute (NHGRI) of the National Institutes of Health (NIH) launched the “$1000 Genome” project (Spencer, 2010) to develop revolutionary sequencing technologies that would enable a mammalian-sized genome to be sequenced for $1000 or less. The gold standards (NHGRI, 2004) are summarized as: (1) high accuracy (less than 1 error in 10,000 bases), (2) long read length (essentially no gaps), (3) high throughput, and (4) low cost (= $1000/genome). With the support from NHGRI and private sectors, massively parallel sequencers based on various technicalities have been released to the market. These instruments are widely called the next-generation sequencers. More specifically, Schadt and coworkers had categorized them into 3 generations (Schadt et al., 2010), i.e., Sanger sequencing—the first-generation, amplification-based massively parallel sequencing—the second, and single-molecule sequencing—the third. This is highly recommended for its clarity.

The 2nd-generation (2nd-gen) sequencing exploits amplification of target DNA and massively parallelized chips, including arrays of microbeads (Roche and Life Technologies/Thermo Fisher Scientific), DNA nanoballs (Complete Genomics/Beijing Genomics Institute), and DNA clusters. Although the 2nd-gen sequencing cost for consumables is approaching $1000 per human genome (Heger, 2014b), it remains challenging to simultaneously attain the four gold standards. Even at a cost for $1000 per genome, it is still too expensive in term of routine test in a hospital setting. High accuracy is currently acquired by replicative sequencing. For read length, most of the 2nd-gen sequencers can sequence only a few hundred bases because of gradual intermolecular dephasing among DNA clones within sequencing reaction sites (Erlich et al., 2008). FLX system is comparable to Sanger sequencers in terms of read length, but it will be phased out by mid-2016 (A Genomeweb Staff Reporter, 2013c). One of the two major breakthroughs brought by the 2nd-gen sequencers is high throughput. For instance, HiSeq X Ten, newly released by Illumina, can daily produce data equivalent to about 7 human genomes at 30 × coverage (Heger, 2014b). However, a sequencing run takes 3 days. Turnaround time per run (TTR) is particularly crucial in genetic test and treatment for newborns (Saunders et al., 2012). Thus, TTR within minutes or even hours is still our imagination. To overcome these problems, it is desperate to develop new sequencing technologies.

Technologies to sequence DNA at the single-molecule level, i.e., the 3rd-gen sequencing, have been anticipated to resolve most, if not all, of the above problems. In these approaches, the error-prone amplification step is eliminated during sample preparation. This category consists of a series of techniques, including nanopore sequencing (Branton et al., 2008), stepwise single-molecule sequencing by synthesis (SBS) (Harris et al., 2008), single-molecule real-time SBS (Eid et al., 2009), single-molecule motion sequencing (Greenleaf and Block, 2006; Ding et al., 2012), electron microscopy (Bell et al., 2012), molecular force spectrometry (Cheng et al., 2012), sequencing by tip-enhanced Raman scattering (Bailo and Deckert, 2008; Treffer et al., 2011), etc.

Among those technologies, nanopore sequencing has been expected to potentially accomplish the gold standards. In 1996, Deamer, Branton and coworkers reported on DNA translocation through α-hemolysin nanopore (Kasianowicz et al., 1996). This seminal work has ushered in the new era of nanopore sequencing. Here we discuss the advances, remaining obstacles, and potential of nanopore sequencing to achieve these gold standards. Finally, we will try to refine the definition of the four gold standards.

## Sequencing by detection of ionic current blockage

### The conception of nanopore sequencing

The idea of nanopore sequencing was proposed by Deamer and Branton and independently by Church (Pennisi, 2012). The concept is that if bases could induce different ionic current bursts during DNA traversing through a tiny channel, then it would become a totally new sequencing technique. In 1993, Deamer, Branton, and Kasiannowicz employed α-hemolysin (α-HL), a toxic pore-forming protein secreted by *Staphylococcus aureus* to attack a lipid bilayer, to detect DNA translocation through α-HL nanopore (Song et al., 1996). In 1996, their results of DNA translocation through α-HL nanopore was published (Kasianowicz et al., 1996).

**Figure 1:**
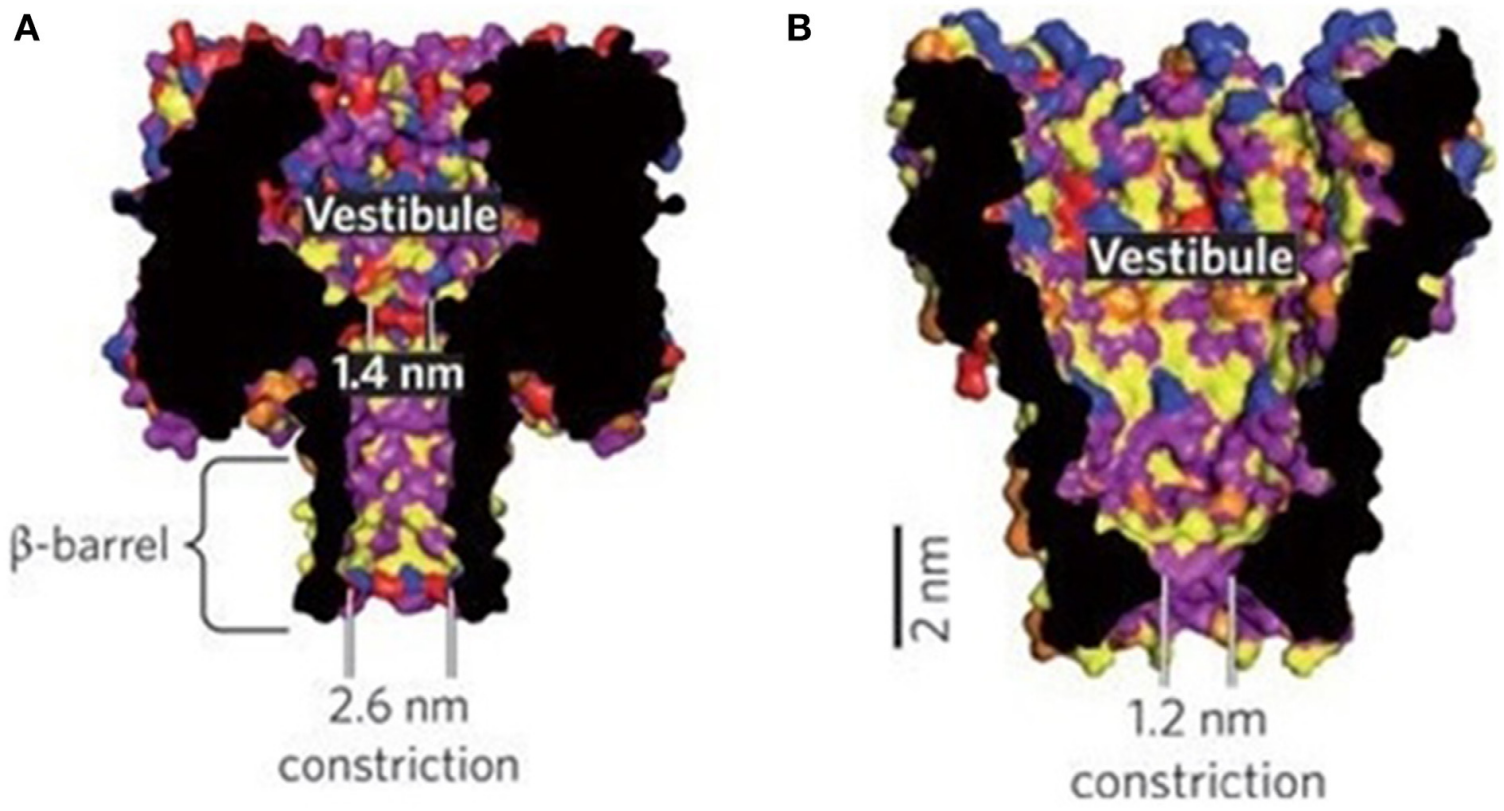
**Nanopore structures of α-HL (A) and MspA (B) (Venkatesan and Bashir, 2011)**. Reproduced by copyright permission of Nature Publishing Group.

Bayley and colleagues reported that α-HL is a 232.4 kDa membrane channel protein (Gouaux et al., 1994). Their crystal structure analysis of α-HL revealed a ~10 nm-high hollow mushroom-shaped homoheptamer complex containing a ~10 nm-wide extramembranal cap and a ~5.2 nm-long transmembrane β-barrel stem (Song et al., 1996). The minimum diameter at the constriction site of the channel is ~1.4 nm, which is connected to the β-barrel with the vestibule of 2.6 nm in diameter at the trans side (Figure 1A).

**Figure 2:**
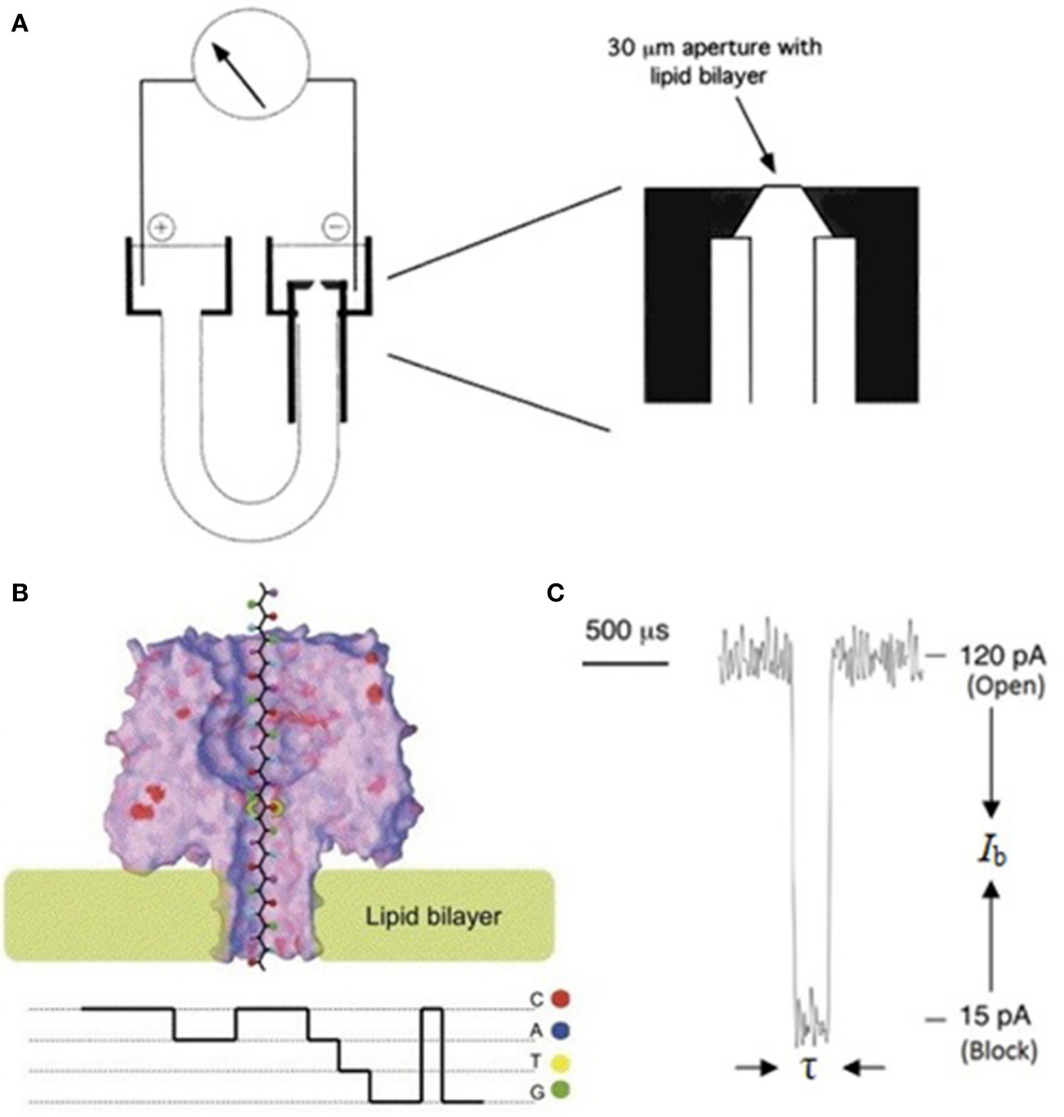
**Schematic illustration of a nanopore sequencing device**. **(A)** A U-tube supports the lipid bilayer membrane bathed in electrolyte solution in which a 120 mV bias is applied. During DNA translocation through the nanopore, ionic current is recorded by a PCA connected to the *cis* (negative) and *trans* (positive) chambers. **(B)** When an ssDNA molecule traverses through α-HL from *cis* to *trans* chamber, the open pore current drops to four different levels (*I*_b_: current blockage), each for a certain time (τ: dwell time). **(C)** Current signals could reveal sequence information (Deamer and Branton, 2002; Bayley, 2006). Reproduced by copyright permissions of American Chemical Society and Elsevier.

An α-HL nanopore is inserted into a lipid bilayer which separates small-volumed chambers, each connected to a cathode and an anode of a patch clamp amplifier (PCA). The ~1.4 nm constriction of α-HL pore allows only individual single-stranded DNA (ssDNA) or RNA other than 2 nm-thick double-stranded DNA (dsDNA) to traverse through. Different bases along the negatively charged DNA strand will cause electric current fluctuations in the course of translocating through the nanopore under an applied electric field. If the fluctuations are base-specific, these electric signals or signatures can be eventually converted into DNA sequence information (Deamer and Branton, 2002; Bayley, 2006) (Figure 2).

### Main challenges and advances in nanopore sequencing

#### Single base resolution

***Single base resolution by protein nanopores***. A fundamental requirement in nanopore sequencing relies on fine pore geometry to acquire single base resolution (SBR). In addition to the constriction site, the β-barrel of α-HL nanopore is ~5 nm long (Figure 1A), capable of accommodating ~10 bases (Wilson et al., 2009; Cherf et al., 2012). Some parts along the barrel are narrow enough to induce additional signals or noise. Indeed, demonstrated by Bayley's group, such a channel has 3 recognition sites (Stoddart et al., 2009) whose interactions would result in more indirect signal interpretations (Purnell and Schmidt, 2009; Stoddart et al., 2010b). MinION, a cellphone-sized nanopore sequencer released to early-access users from Oxford Nanopore Technologies (ONT), uses base calling algorithms to decode the ionic current blockage, which is not a function of single base but hexamer (Heger, 2014a), despite read length and rate up to 50 kilobases (Kb) and 100 bases/s, respectively, are groundbreaking (A Genomeweb Staff Reporter, 2013b; Heger, 2014a; Karow, 2014d). Base calling algorithms need to be improved to reduce error rates (Heger, 2014a). Another possible alternative, suggested by Jaffe, is to perform multiple sequencing using different types of engineered nanopores (Heger, 2014a). To eradicate error rate problem, Bayley et al. proposed to eliminate one or two recognition sites through genetic engineering, and demonstrated its feasibility (Stoddart et al., 2010a). Recently, Ervin and colleagues reported that these excess recognition sites were eliminated through site-directed mutagenesis, enabling base sensing at single recognition site (Ervin et al., 2014). More accurate SBR could be anticipated from these engineered nanopores.

In 2004, Schulz's group reported that an octameric membrane porin, secreted by *Mycobacterium smegmatis*, formed a funnel-shaped MspA nanopore with an outlet of 1.2 nm in diameter (Faller et al., 2004) (Figure 1B). Gundlach's group demonstrated that MspA pore had great potential to sequence DNA (Derrington et al., 2010). The feature of single recognition site of MspA pore seems to be more advantageous than α-HL (Manrao et al., 2011). This group replaced negatively-charged amino acids with neutral asparagine residues in pore's constriction site, and with positively charged basic residues in pore's entrance through genetic engineering (Butler et al., 2008), which enabled easy DNA capture and deceleration of DNA translocation through the pore. They later demonstrated that the engineered MspA pore exhibited better base resolution than α-HL pore by generating larger signal difference between bases (Derrington et al., 2010). However, development of new methods is needed to avoid signal overlapping between different bases, particularly deoxynucleotides adenine and guanine (Derrington et al., 2010; Manrao et al., 2011), to increase accuracy. For precise SBR, the length of the recognition region of a nanopore shouldn't be larger than ~0.5-nm, equivalent to phosphorus-phosphorus distance of a nucleotide (or base spacing) in an ssDNA strand (Wilson et al., 2009; Cherf et al., 2012). The constriction region of MspA is about 0.6 nm long (Manrao et al., 2012), which means signal interference from adjacent bases (Manrao et al., 2011, 2012; Laszlo et al., 2013, 2014). Their previous work showed that about four bases together around the constriction region contribute to the overall current blockage (Manrao et al., 2011, 2012). Recently, they have resorted to tetramer maps, which are the standard electric signal curves collected by measuring current blockage signals when each of the combination of 256 possible 4-mers is translocating through the pore, and algorithms to circumvent this problem (Laszlo et al., 2014).

***Single base resolution by graphene and other solid-state nanopores***. Besides α-HL, researchers have long been investigating solid-state nanopores, with an initial hope to tackle problems in the protein nanopores, such as instability and dimension-tuning difficulties. In 2001, Golovchenko, Branton and colleagues demonstrated that nanopores as small as 1.8 nm in diameter could be fabricated on Si_3_N_4_ membrane using focused ion beam (FIB), and found that pores could be opened and closed below 4°C and above 5°C, respectively, a critical phenomenon for diameter tuning (Li et al., 2001). Since then, solid-state materials have attracted great attention because of their excellent mechanical and thermal stability, selectable chemistry, nano-fabrication, and electronic integration choices. In 2003, Dekker's group showed that fabrication of nanopores on SiO_2_ wafer could be controlled at the single-nanometer precision using focused electron beam (FEB) (Storm et al., 2003), which has been widely adapted (Dekker, 2007). In the meantime, a variety of materials for fabrication of solid-state nanopores by different technologies have been reported, including organic polymer (Siwy and Fuliñski, 2002), single-walled carbon nanotubes (SWCNT) (Liu et al., 2010, 2013a; Wang and Huang, 2010), glass (Sha et al., 2013; Li et al., 2013c), hafnium oxide (Larkin et al., 2013), boron nitride (Liu et al., 2013b), etc.

Although the diameter of silicon-based nanopores (SBNs) can be controlled at the nanometer or even single Angstrom precision (Storm et al., 2003; Chen et al., 2004; Kwok et al., 2014; Yanagi et al., 2014), plentiful experiments have revealed that solid-state nanopores have several drawbacks (Dekker, 2007), for example, structural irregularity, large pore length, and poor repeatability. Irregular geometry would cause larger current noise. Control of pore length is becoming one of the most serious issues. A question arising from the results of over a dozen-year SBN studies is whether or not SBNs' length can be shortened to the sub-nanometer scale in order to identify single bases along an ssDNA strand. Early work of Li and colleagues showed that SBN length normally ranged from 8 to 20 nm, depending on ion beam species (Cai et al., 2006). Up to date, the shortest effective pore length has reduced to 1.7 nm (Venta et al., 2013). Based on the fact of 0.5 nm base spacing (Wilson et al., 2009; Cherf et al., 2012), a nanopore with 1.7-nm effective length can accommodate ~4 consecutive bases which confuse SBR. Although decoding of signals generated by the context of a base at the recognition site shared by its surrounding partners is possible (Stoddart et al., 2010b; Laszlo et al., 2014), unambiguous base identification is compromised. Because the goal is to identify a single base at the recognition site, thinner membranes are preferable. Recently, Hall's group reported that they fabricated nanopores on 1.4-nm thick membrane using Helium ion beam (Suk and Aluru, 2014). According to Wanunu et al. (2010b), the effective length of sandglass-shaped pores is about one third of the membrane thickness, on which the pore is drilled. A nanopore fabricated on membrane of 1.4 nm thick has a 0.47-nm effective pore length, which is comparable to base spacing. SBR using such pores needs further investigation. For precise SBR, an ideal effective nanopore length should be less than half the base spacing. Although it is still an open question for such an effective pore length to completely nullify neighboring bases' contributions to overall current blockage in the nano-scaled niche, this short effective pore length can at least minimize the neighboring effects. In the meantime, molecular dynamic (MD) simulation can also be helpful to address this issue. It is therefore necessary to explore new materials/methodologies (Xu et al., 2013a; Sawafta et al., 2014) while thinning membranes for fabrication of SBNs (Marshall et al., 2012; Suk and Aluru, 2014).

Graphene, an ultrathin and impervious membrane, has attracted growing interests because of its excellent conductivity, atomic thickness, and robust mechanical stability (Novoselov et al., 2004; Meyer et al., 2007). In 2008, Drndić's group reported fabrication of nanopores in suspended graphene sheets using FEB (Fischbein and Drndić, 2008). In 2010, three groups demonstrated that graphene has a great potential in DNA sequencing (Garaj et al., 2010; Merchant et al., 2010; Schneider et al., 2010). The thickness of a single-layer graphene is only 0.335 nm (Novoselov et al., 2004). Garaj et al. reported that monolayer graphene nanopore has exceptionally high diameter sensitivity of over 0.65 nA/Å (Garaj et al., 2013), and its 0.6-nm insulating thickness in solution leads to the calculated spatial resolution of 3.5Å (Garaj et al., 2010).

These results suggest that graphene nanopores might possibly sequence DNA (Siwy and Davenport, 2010). MD simulation performed by Aksimentiev's group moderately supported its possibility with the prerequisite that the orientation of the bases in the nanopore is precisely controlled (Wells et al., 2012). A recent MD analysis by Wu's group showed similar results (Lv et al., 2014). Another simulation study by Chen's group implied that nanopore in monolayer graphene is less sensitive than that in few-layer graphene (Li et al., 2013a). Experiments on SBR by graphene nanopores are badly needed to answer these questions.

**Figure 3:**
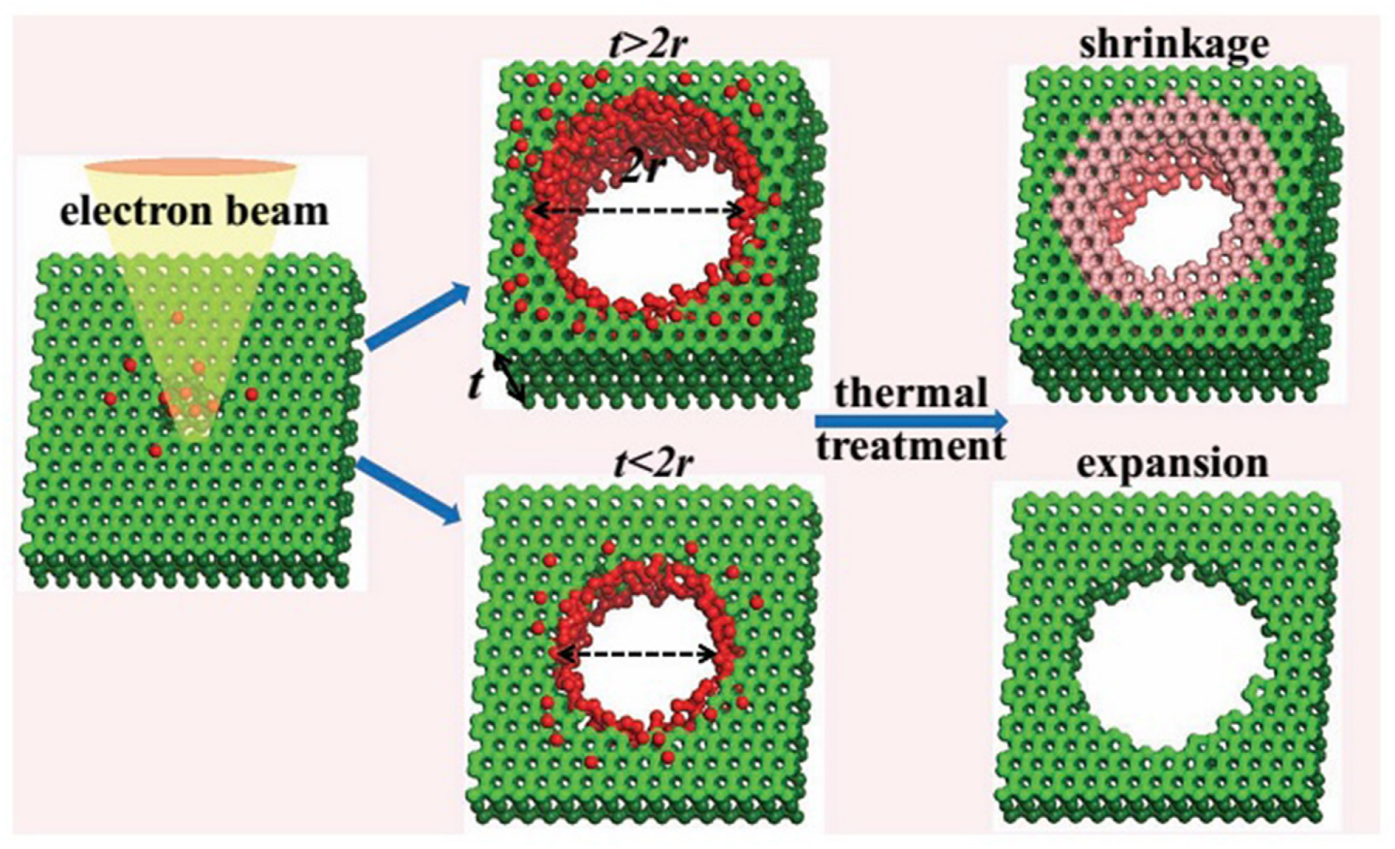
**Schematic illustration of the shrinkage and expansion of graphene nanopore (*t* represents height of graphene, *2r* for nanopore diameter) (Xu et al., 2012)**. Reproduced by copyright permission of John Wiley and Sons.

Furthermore, the structural geometry of these graphene nanopores is usually irregular, which introduces noise if the orientation of the bases cannot be precisely controlled. To improve fabrication technology, it is essential to understand the mechanism of pore formation. In 2009, Zettl's group investigated the dynamics of carbon atoms at the edge of nanopores in a suspended single-layer graphene with the transmission electron aberration-corrected microscope, and found the edge lattice could be repaired at a slower rate than atom erosion (Girit et al., 2009). Banhart's team used FEB of 1-Å diameter to irradiate singly atoms at predefined positions from carbon nanotubes (Rodriguez-Manzo and Banhart, 2009), demonstrating a method for nanopore fabrication at the Angstrom precision. In 2011, Zandbergen's group reported the discovery of an intrinsic self-repair mechanism to keep the graphene in a single-crystalline state during 300 kV FEB etching at 600°C (Song et al., 2011). The next year, Kim's group reported (Lu et al., 2012) that, under a 200 kV FEB, nanopores in multilayer graphene could be completely closed at 400°C. Shrinking of nanopores to smaller diameter (<2.5 nm) and opening of that to larger diameter (≥3 nm) were observed at both 800 and 1200°C. They explained that shrinkage and expansion of the nanopores result from the interplay between two mechanisms, erosion of carbon atoms from the edge of the pore and generation of carbon ad-atoms, where the former facilitates pore expansion but shrinkage by the latter. Sun's group discovered that shrinkage and expansion of nanopores in multilayer graphene could be induced by heating at 400°C without irradiation (Figure 3), and was linearly dependent on the ratio of pore diameter to graphene thickness (Xu et al., 2012). The transition point of the ratio is around 1, where the ratio above 1 result in pore opening, and that below 1 leads to pore closing. They concluded that the knock-out carbon atoms by FEB are necessary for pore shrinkage. The follow-up fabrication of nanopores on free-standing monolayer graphene with 1-Å FEB by Zandbergen's group has shown that processing at 600°C is suitable for pore etching at nearly atomic precision, while temperature at 20°C and 800°C result in contamination and faster self-repair than removal of carbon atoms. Other significant achievements were microsecond-imaging (5–30 μs) without further pore damage, fabrication of array of patterned nanopores, and possibility of automation (Xu et al., 2013b).

These findings will significantly enhance fine diameter-tuning technologies for nanopore fabrication in graphene sheets. However, several challenges still exist in FEB-based nanopore manufacture even if extremely axisymmetric nanopores can be fabricated through removal of carbon atoms one at a time. First, sample capacity of scanning or transmission electron microscopes is currently limited at the square millimeter scale despite production of 30-inch area, predominantly monolayer graphene sheets, has been demonstrated by Ahn, Hong, and coworkers (Bae et al., 2010). Second, FEB procedure is time-consuming. Accordingly, parallelization of graphene nanopores comparable to that of Ion Torrent systems is presently a distant expectation which depends on progress of methodology. Third, as pointed out by Bayley (2010), carbon atoms at the pore's edge may be modified by a number of chemical function groups during their exposure to air and water after FEB irradiation, which might confound base discrimination. Simulation work by Král's group showed that modification of pore's edge with positively charged hydrogens by hydrogenation passivation could facilitate DNA translocation through graphene pores (Sint et al., 2008). In 2013, Dekker's group reported (Schneider et al., 2013) that graphene coated with pyrene ethylene glycol could inhibit graphene-nucleobase interactions caused by π −π stacking. However, the pore length was increased from 1.5 nm to about 5.5 nm by this coating procedure. SBR in this sequencing system becomes a new issue. Recently, Lee, Chisholm, and colleagues used Si to passivate carbon atoms at the edge of the graphene pores (Lee et al., 2014), and demonstrated that these Si-passivated graphene nanopores could be stored for many months under ambient conditions, indicating the feasibility to develop vigorous graphene nanopore sequencers.

In search of new approaches, Russo and Golovchenko demonstrated fabrication of nanopores with diameters at the Angstrom scale in monolayer graphene by inducing defects with energetic ions followed by the edge-selective electron recoil sputtering (Russo and Golovchenko, 2012). This method does not need electron beam to be focused and allows scalable production of patterned nanopore array.

In addition to graphene material, Yu's group reported that fabrication of nanopores with 1.1 nm effective length in double-layer boron nitride membrane by FEB, which provided more sensitive detection of DNA translocation than SBNs (Liu et al., 2013b). However, the frequency of DNA translocation events was not high due to its low hydrophilicity. Recently, Shan, Lu, and colleagues (Zhou et al., 2013) demonstrated that this hydrophilicity could be improved by UV-ozone treatment. Radenovic's group reported (Liu et al., 2014) that they used 0.7 nm-thick monolayer molybdenum disulfide (MoS_2_) to fabricate nanopores, which showed lower membrane-DNA interaction. These new materials provide more options for SBR in the future research. However, it should be beared in mind that some issues for graphene nanopores remain similar to these new species of pores.

#### Speed control of DNA translocation through nanopores

Because of the intrinsic limitations of commercially available PCAs, sampling rate of current measurement is commonly lower than 250 kHz due to signal noise [M. Wanunu had a comprehensive review in this regard (Wanunu, 2012)], except 500 kHz used by Gundlach et al. (Laszlo et al., 2014). Under the effective driving voltage, namely minimum voltage to impel DNA to pass through the pore (Henrickson et al., 2000), DNA translocation speed is too fast (300 bases/ms) (Akeson et al., 1999; Meller et al., 2000) for PCA to identify individual bases. Hence, it is crucial to reduce the speed to about 1 base/ms to allow increment of the number of ions between a purine and an adjacent pyrimidine from ~100 to 100–1000 folds (Deamer and Akeson, 2000) or to develop faster detection electronics without sacrificing signal-to-noise ratio.

**Figure 4:**
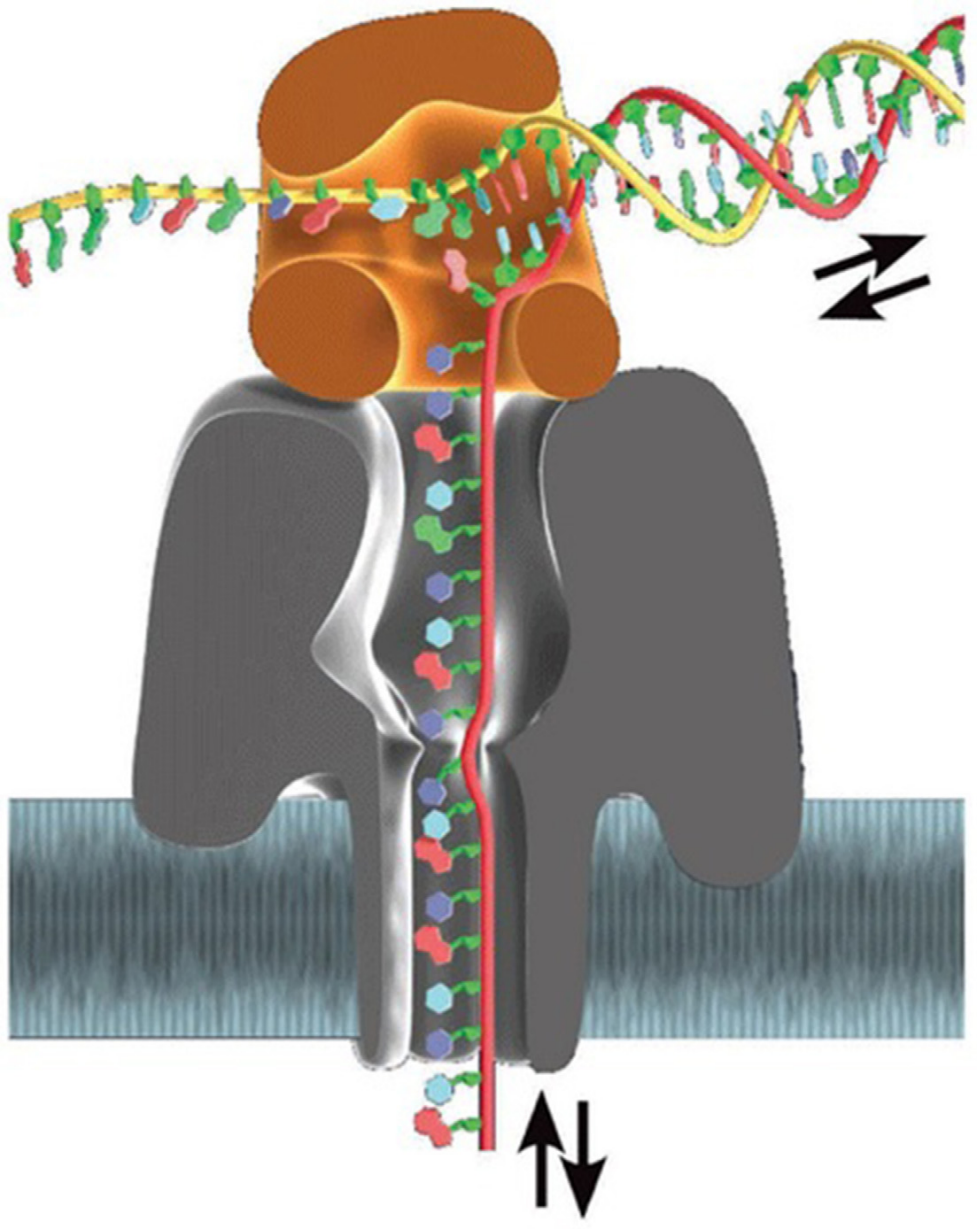
**Base-by-base ratcheting of ssDNA out of α-HL nanopore by Φ29 DNA polymerase (Schneider and Dekker, 2012). Reproduced by copyright permission of Nature Publishing Group**.

Several groups have endeavored to develop efficient approaches to slow down DNA translocation speed. Over a dozen of control methods have been published since the first attempt by Li's team (Fologea et al., 2005). Many of these techniques showed substantial improvements in speed control. Although each of the above has its own pros and cons, the most significant progress has resided in ratcheting of DNA molecules by various ways, in particular molecular motors (Figure 4).

In 1989, Church and colleagues conceived that DNA polymerase could be an ideal motor to precisely control DNA translocation through nanopore (Church et al., 1998). Afterward, Ghadiri's group performed real-time observation of ratcheting dynamics of a few DNA polymerases while they pulled ssDNA out of α-HL nanopores base by base during primer extension, demonstrating that DNA polymerases are hopeful candidate motors (Cockroft et al., 2008; Chu et al., 2010). Most intensive work in this aspect comes from Akeson and colleagues. They have reported a series of investigations on dynamics of enzyme-DNA interactions since 2007 (Benner et al., 2007; Hornblower et al., 2007). Stability of the motor-DNA complex under the nanopore sequencing condition is one of the most important parameters because it determines read length and accuracy. In this case, an ideal motor would be an enzyme with extremely long processivity, in addition to its potent activity (as long as it is not faster than a PCA's temporal resolution), which is together translated into sequencing rate. They found that, at 80 mV, T7 DNA polymerase could synthesize DNA while *E. coli* Klenow fragment couldn't, and easily dissociated from DNA at the voltage bias between 165 and 180 mV (Benner et al., 2007; Gyarfas et al., 2009; Wilson et al., 2009; Olasagasti et al., 2010). Later they reported that processive Φ29 DNA polymerase could catalyze DNA synthesis against a 180-mV bias (Lieberman et al., 2010). They further demonstrated that this enzyme could synthesize DNA at an averaged rate of 2.5–40 bases/s though its processivity of several tens of Kb (Blanco et al., 1989) is still needed to be validated under such conditions.

This Φ29 motor has been also introduced into MspA nanopore system (Manrao et al., 2012). This nanopore technique has been exclusively licensed to Illumina (A Genomeweb Staff Reporter, 2013a). Recently, Gundlach's team reported that the motor generates read length up to 4.5 Kb when it is used to sequence phi X genome (Laszlo et al., 2014). However, the fact that read length distribution is much shorter than the library sample indicates that dissociation between the enzyme and DNA is substantial (Laszlo et al., 2014). Early, ONT disclosed that MinION sequencer produces read length exceeding 50 Kb (Karow, 2014d), and an 8.5-Kb read by MinION has been posted by Karow (2014a); Loman et al. (2014). Although read length needs to be further elongated, both cases are impressive compared with the 2nd-gen sequencers, suggesting a breakthrough toward one of the 4 gold standards and possibility for single-cell sequencing without *in vitro* genome amplification that erases epigenomic information. But error rate of both sequencing systems is still high. Gundlach et al. explained that errors are induced by positional switches of DNA bases in pore's recognition site due to the stochastic motion of the motor, DNA-pore interactions, and deviations from their tetramer maps (Laszlo et al., 2014). Therefore, obtaining of high quality *de novo* sequencing in these sequencers is still challenging.

Beside ratcheting DNA with enzymes, Li's team reported a successful control of DNA threading through nanopores at a speed below 1 nm/ms using piezoelectrics-based method (Hyun et al., 2013), which is slow enough for base identification. Recently, Timp's group has reported that DNA velocity can be controlled at 1.0 nm/s against a 0.45 V applied field using the tip of atomic force microscope (AFM), and found that “stick-slip” motion coexists with frictionless sliding (Nelson et al., 2014) (see Section Improvements in Detection Electronics).

Stolovitzky and colleagues designed a “DNA transistor” (Polonsky et al., 2007; Luan et al., 2010, 2011), a solid-state nanochannel in which an anode ring flanked by two cathode rings are embedded, to trap a DNA strand while pulling one end of it out of the channel using a “harmonic spring” such as AFM or optical tweezers. Their simulation results showed that this device could ratchet DNA with a constant speed at the Angstrom scale (Luan et al., 2010). Recently, they published a paper on their fabrication of DNA transistors using reactive ion etching (RIE) (Bai et al., 2014). The mean diameter of the resultant nanopores is around 18 nm. Preliminary tests on a nanochannel of 20-nm in length showed that the transistor is unable to decelerate DNA speed. They believed that its diameter is too large. Nevertheless, the advantage is that RIE enables them to fabricate nanopore arrays at the wafer scale.

Another possible alternative is to stretch DNA strand by tethering both ends on piezo-controlled tips, as demonstrated with optical tweezers by Bustamante's group (Moffitt et al., 2006). However, single-molecule manipulation in such devices is very difficult. Its unique advantage is that the base spacing can be expanded from 0.5 to 0.75 nm (Smith et al., 1996), enabling nanopores to distinguish single bases at an elevated precision. The major challenges, however, are to increase a dynamic range that piezoelectronics can be precisely controlled and operate DNA molecules in arrayed nanopores.

#### Improvements in detection electronics

Fabrication of arrays of protein and solid-state nanopores has already been demonstrated (Kim et al., 2006; Hall et al., 2010). In the meantime, one should consider how to use compatible PCAs for simultaneous signal recording. Features of an ideal PCA include high signal-to-noise ratio, high bandwidth, and multiplexing detection.

In 2006, based on a complementary metal oxide semiconductor (CMOS) circuit, two groups reported their fabrications of integrated PCAs with sensitivity in the picoampere range and a bandwidth up to 10 MHz in sub-micron CMOS processes (Laiwalla et al., 2006; Zhu et al., 2006). Later, Collins and colleagues reported on the test of a 6 integrated amplifier, demonstrating its capability for multiplexing detection (Gierhart et al., 2008). Culurciello, Sigworth and colleagues also introduced resistive compensation circuit and a parasitic capacitive compensation circuit to compensate the capacitance and resistance of the electrode, respectively (Weerakoon et al., 2009, 2010). Furthermore, Dunbar and coworkers have reported that a highly-sensitive amplifier is fabricated in a 0.35 μm CMOS process (Kim et al., 2011, 2013a), and used this device to test DNA translocation through α-HL nanopore, demonstrating its potential for accurate detection of abasic sites (Kim et al., 2013a,b). They have also reported that the size of feedback resistor is successfully reduced by a factor of 10 via introduction of a novel pseudo-resistor technique, which is an important step toward miniaturization (Kim et al., 2013c). Further improvement to reduce noise is under investigation as well (Kim et al., 2013b). Another group, Shepard and colleagues, has been developing high-bandwidth electronic interfaces for fast temporal resolution (Anderson, 2012). They reported that a new CMOS preamplifier for an array of SBNs has a signal-to-noise ratio >5 at 1 MHz sampling rate (Rosenstein et al., 2012). Calculation results showed that sampling rate at 40 MHz is possible (Rosenstein and Shepard, 2013).

A commercial multichannel PCA for parallel detection has been demonstrated by Behrends' group (Baaken et al., 2011). Other nanopore sequencing companies, including ONT (Davies, 2012), Illumina (A Genomeweb Staff Reporter, 2013a), and Genia (Toner, 2012), which has been acquired by Roche (Karow, 2014e), are all devoted to develop their own integrated circuit platforms for multiplexing detection. ONT has achieved parallelization of 512 pores in MinION sequencer (Heger, 2014a), and is heading to an array of 100,000 pores in PromethIon (a more powerful version than MinION) (Karow, 2014c). Apparently, Electronic Bio Sciences, which is developing a sequencing platform using engineered α-HL nanopre with a single recognition site (Karow, 2009; Ervin et al., 2014), may be involved in the same endeavor as well.

## Other nanopore sequencing

### Methods that circumvent the base spacing limit

The Å-scaled base spacing limit has long been challenging direct base recognition by threading DNAs through a pore. A few groups have proposed to develop new pore-related technologies to circumvent this problem. One of the answers is to artificially enlarge base's spacing, one way or another.

#### Exonuclease-assisted nanopore sequencing

To overcome signal interference between neighboring bases, we and Bayley's group have independently proposed a new technique, i.e., exonuclease-assisted nanopore sequencing, which could also detect 5-methyl-2′-deoxycytosine (5-mdC) besides the 4 regular bases. The detection setup is the same as the above threading method. The conception is that different 2-deoxyribonucleoside 5′-monophosphates (dNMPs) induce distinct electrical signatures while they are traversing through a nanopore. When individual dNMPs are cut off from one end of a DNA molecule by an exonuclease, and sequentially traverse through a nanopore under an applied voltage, the sequence information of the target DNA molecule can be acquired from the electrical signals.

**Figure 5:**
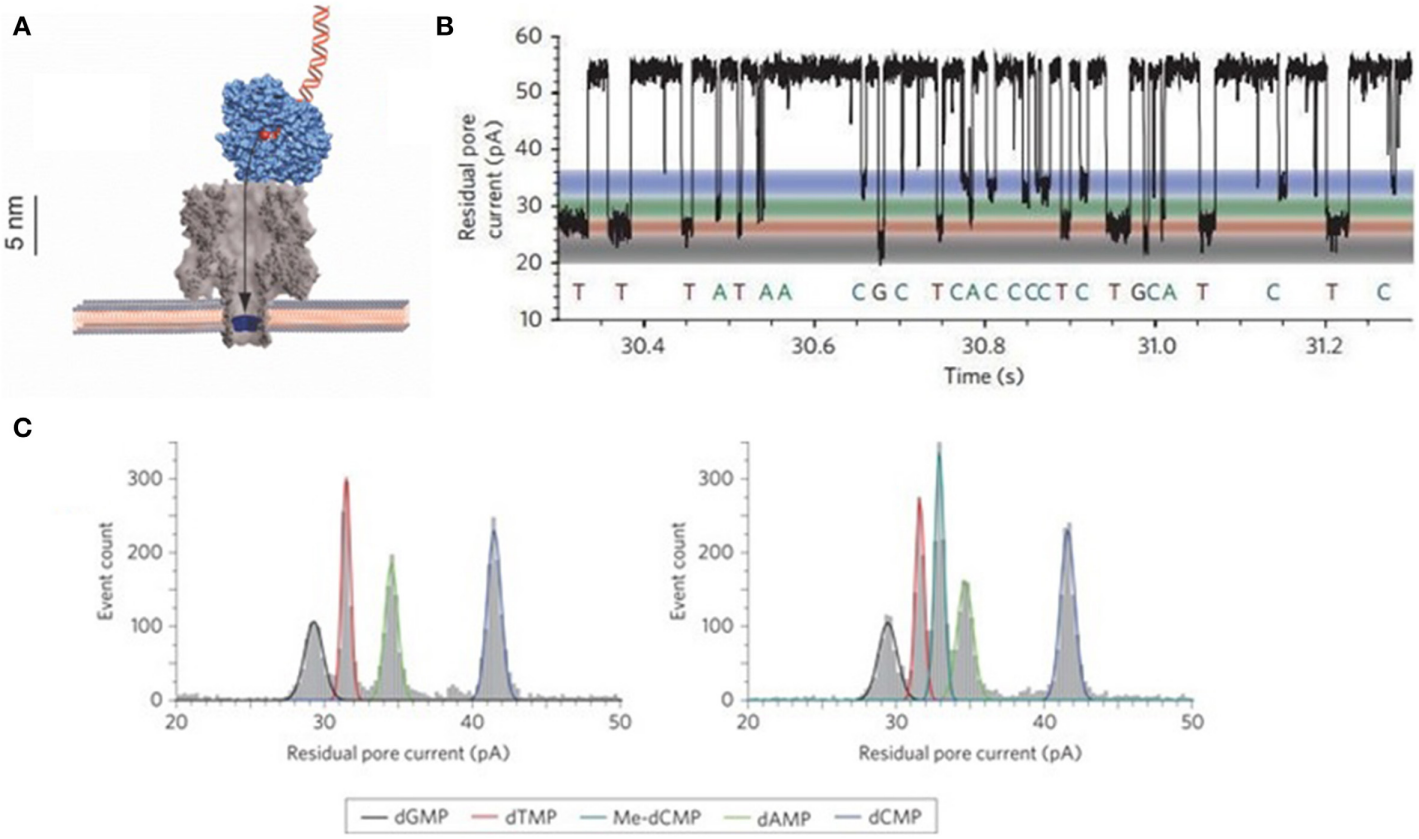
**Schematic illustration of exonuclease sequencing**. **(A)** Illustration of exonuclease bound onto α-HL. **(B)** Individual nucleotides translocation events. **(C)** Detection of 5-mdC or Me-dCMP and dNMPs through α-HL nanopore (Clarke et al., 2009; Reiner et al., 2012). Reproduced by copyright permissions of American Institute of Physics and Nature Publishing Group.

The regimen suggested by Bayley's group is illustrated in Figure 5. This group has proved a mean accuracy of 99.8% for four dNMPs and the ability of 5-mdC identification by engineering α-HL nanopore to facilitate longer dwelling time for accurate detection (Astier et al., 2006; Clarke et al., 2009). Recently, this group also demonstrated the possibilities of sequencing RNA and detecting RNA uridylation at their 3′ ends by α-HL nanopore (Ayub and Bayley, 2012; Ayub et al., 2013; Clamer et al., 2013; Cracknell et al., 2013). The RNA-sequencing expands potential application of nanopore into direct sequencing of transcriptome and detecting of post-transcriptional RNA modification at the single-molecule level. Additionally, selective detection of microRNAs has been demonstrated independently by Wanunu et al. (2010b), and Gu's group (Tian et al., 2013).

Unlike the protein pore, Wang et al. proposed SWCNT pores to detect dNMPs and improved accuracy through adjusting pore diameter and length (Wang, 2010; Wang and Huang, 2010). A handful of hurdles exist in this system, e.g., nanopore fabrication, fine-tuning of the pore dimensions, and among others. SWCNT nanopores have been fabricated using nanotechnology by Lindsay, Nuckolls and coworkers (Liu et al., 2010), and via inserting ultrashort SWCNT into lipid bilayers by Wu's group (Liu et al., 2013a), which could be adapted to our sequencing system to overcome the hurdles.

#### NanoTag-SBS sequencing

**Figure 6:**
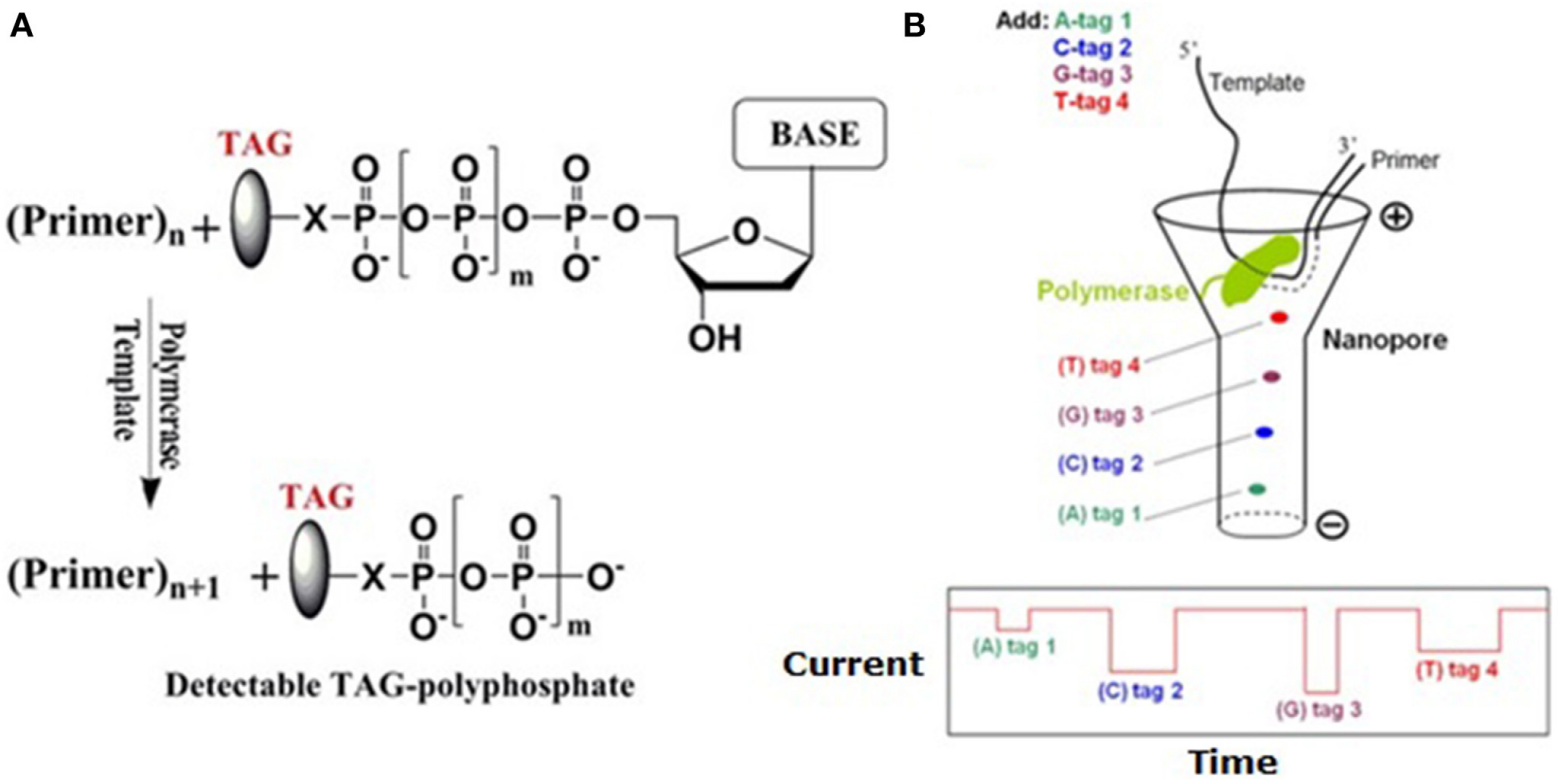
**Schematic illustration of Nano-SBS**. **(A)** Tag-phosphate is cleaved off when the nucleotide is incorporated into growing primer. **(B)** During SBS, these tags are sequentially released and driven through the nanopore (upper), where they induce unique current blockage signals (lower) (Kumar et al., 2012). Reproduced by copyright permission of Nature Publishing Group.

Ju and colleagues, who developed a 2nd-gen SBS method (Seo et al., 2005; Wu et al., 2007; Guo et al., 2008), reported a nanotag-based real-time sequencing by synthesis (NanoTag-SBS) (Kumar et al., 2012), which is different from zero-mode waveguide-based PacBio RS platforms (Eid et al., 2009). In this system (Figure 6), a polymerase is immobilized in the vicinity of pore's *cis* entrance. During DNA synthesis, four 2-deoxyribonucleoside 5′-triphosphates (dNTPs), each labeled with one of the 4 tags carrying poly (ethylene glycol) repeats of different lengths, is incorporated into the growing strand according to Watson-Crick rule. The released diphosphate-carrying tags would sequentially traverse through the pore under a voltage and induce distinct current blockage signals, which will be translated into sequence information. This Nano-SBS has been licensed to Genia Technologies (Karow, 2012), a company for development of α-HL pore arrays on semiconductor chips. In this system, attachment of one polymerase onto one pore's entrance is extremely challenging. Karow reported that Church's group, which developed the 2nd-gen sequencing by ligation, is going to complete this task through protein fusion technology (Shendure et al., 2005).

#### Optipore

Except for the enhancement of signal-to-noise ratio by nanopore structure during optical detection (Chansin et al., 2007; Hong et al., 2008), the other advantage of combining nanopores with optical methods lies on the simultaneous probing of DNA translocation signals (Sawafta et al., 2014).

**Figure 7:**
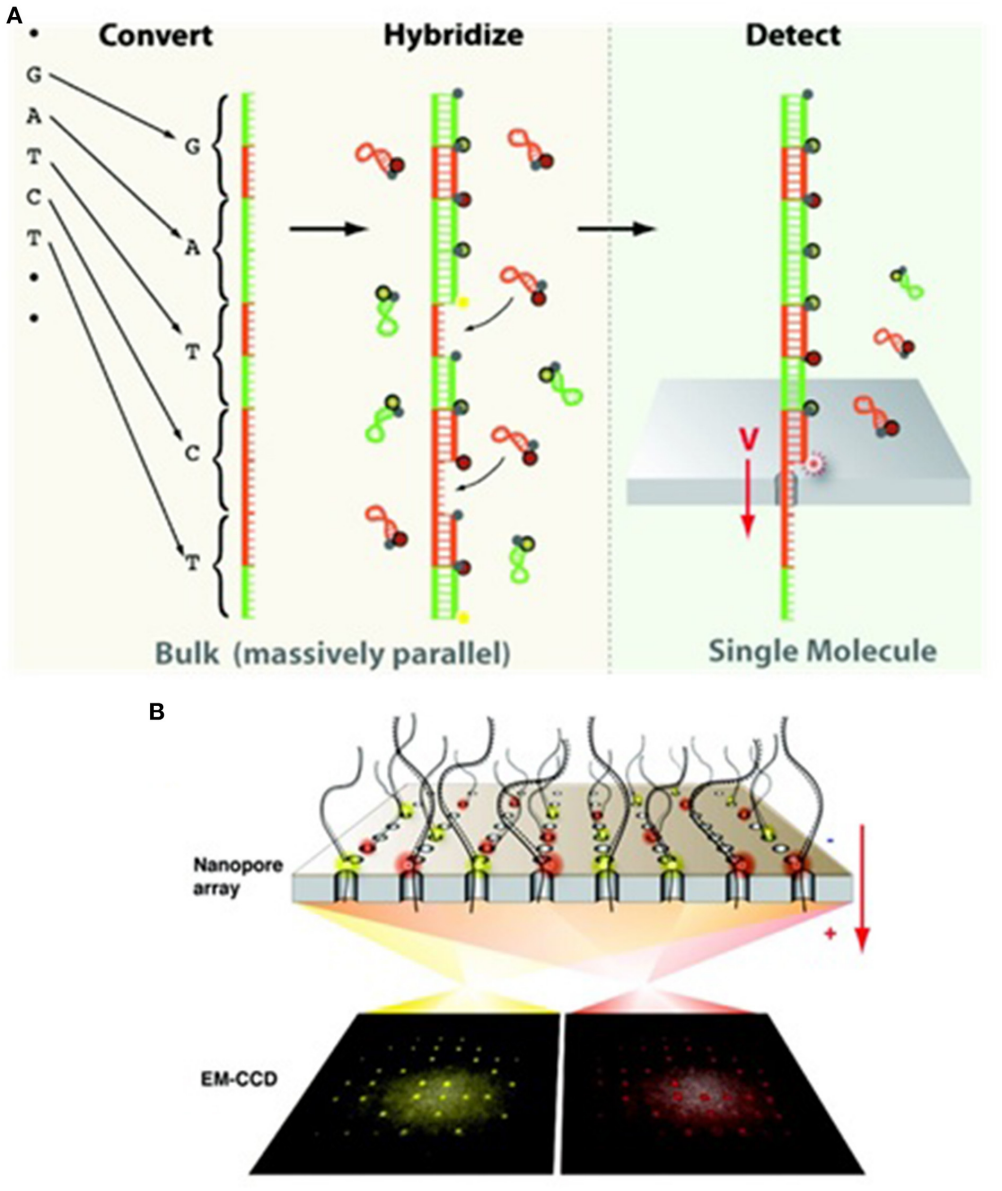
**Schematic illustration of the optipore sequencing**. **(A)** Target ssDNA fragments are converted to predesigned oligonucleotides (left), hybridized to probes carrying molecular beacons (middle), and detected by EMCCD while traversing through a nanopore (right). **(B)** The sequencing scheme of the parallelized optipores (McNally et al., 2010). Reproduced by copyright permission of American Chemical Society.

Meller's group developed a novel optipore sequencing method (Soni and Meller, 2007; McNally et al., 2010). In this system, each nucleotide on a target ssDNA is converted into a binarily-coded oligonucleotide sequence, i.e., the designed DNA polymers (DDP) (Figure 7A left). In other word, each of the four converted binarily-coded oligonucleotides contains two unique sequences, designated “0′ and “1.” For example, base A, G, T, and C are represented by “11,” “10,” “01,” and “00,” respectively. Four probes complementary to the four converted bases are differentially labeled with four different fluorescent dyes, or molecular beacons, at their heads to encounter nanopore first and a universal quencher at their tails (Figure 7A middle). In addition, these probes are so designed that they are capable of forming hairpins through intrastrand hybridization without competition with the coded complementary oligonucleotides. Therefore, fluorescent dyes can be quenched before they are hybridized with DDP. After hybridization, all beacons of the probes except the leading one are quenched by the neighboring quenchers. Hybridized DDPs with a distinct single-stranded overhang are loaded in *cis* chamber. The overhangs can lead DDPs to enter an array of 1.5-nm solid-state nanopores. When the beacons at the leading probes reach the pore entrances, they can be excited by a laser beam, and their emission can be recorded by electron multiplying charge coupled device (EMCCD) (Figures 7A,B right). Because these pores only allow ssDNA to thread (Figure 7A right), probes at the pore's entrances can be unzipped off (Sauer-Budge et al., 2003; Mathé et al., 2004; McNally et al., 2008) and self-hybridized, and the beacons are subsequently quenched. Due to its inherently small size, this system is amenable to parallelization (Torre et al., 2012). NobelGen predicted that a nanopore of the DDP could read over 100 bases/s (Karow, 2010). An array of 400 × 400 pores would finish a human genome within 30 min. TTR, including sequencing with read length over 200 bases and assembly with a clinical grade accuracy, would be within 24 h at a cost for much less than $2000 (Karow, 2011).

#### Hybridization-based sequencing

Hybridization-assisted nanopore sequencing (HANS) was conceived by Ling et al. (2006), who cofounded NABsys, Inc. in 2004. In this scheme, copies of target ssDNA are hybridized with each of a set of short oligomer (initially hexamer) library to generate hybrid molecules containing hybridized sites (dsDNAs). When the dsDNA sites pass through a nanopore, they induce stronger ionic current blockage than their ssDNA counterparts. Therefore, a stronger blockage signal is an indication of the local target DNA sequence complementary to the known hexamer sequence. When a hybrid molecule passes through a nanopore, it generates a distinct linear map of dsDNA-ssDNA distribution. Finally, these maps are aligned up by software to generate sequence information of the target ssDNA. This method is readily for massive parallelization. The most impressive feature is that it circumvents the demand for SBR. In 2010, they demonstrated that ssDNA and dsDNA in such a hybrid molecule could be identified (Balagurusamy et al., 2010). The pivotal requirement for HANS is that each hybrid molecule should pass through a nanopore at least at its own constant speed so that accurate assembly could be obtained.

#### Sequencing by expansion™

Based on the same core idea of magnifying signals from individual bases, Stratos Genomics is dedicated to the development of “Sequencing by Expansion™” (SBX) method (Karow, 2014f), where individual bases along ssDNA chain are converted into a 50-fold larger surrogates called Xpandomers, which could generate detectable signals. The sequence is then read out as the Xpandomers sequentially when passing through a nanopore array. It was reported that read length of the SBX method reached 210 bases in 2013. However, technical details about the base conversion are not currently available.

### Innovative nanopore sequencing methodologies

The ionic current detection system is based on Coulter counter (Wanunu, 2012). The current fluctuations are conventionally monitored by PCA's headstage. However, detection models have diversified along with the fast development of nanopore-related sequencing technologies during the last decade. In this category, different detection methods are discussed.

#### Sequencing by electronic tunneling (SBET)

**Figure 8:**
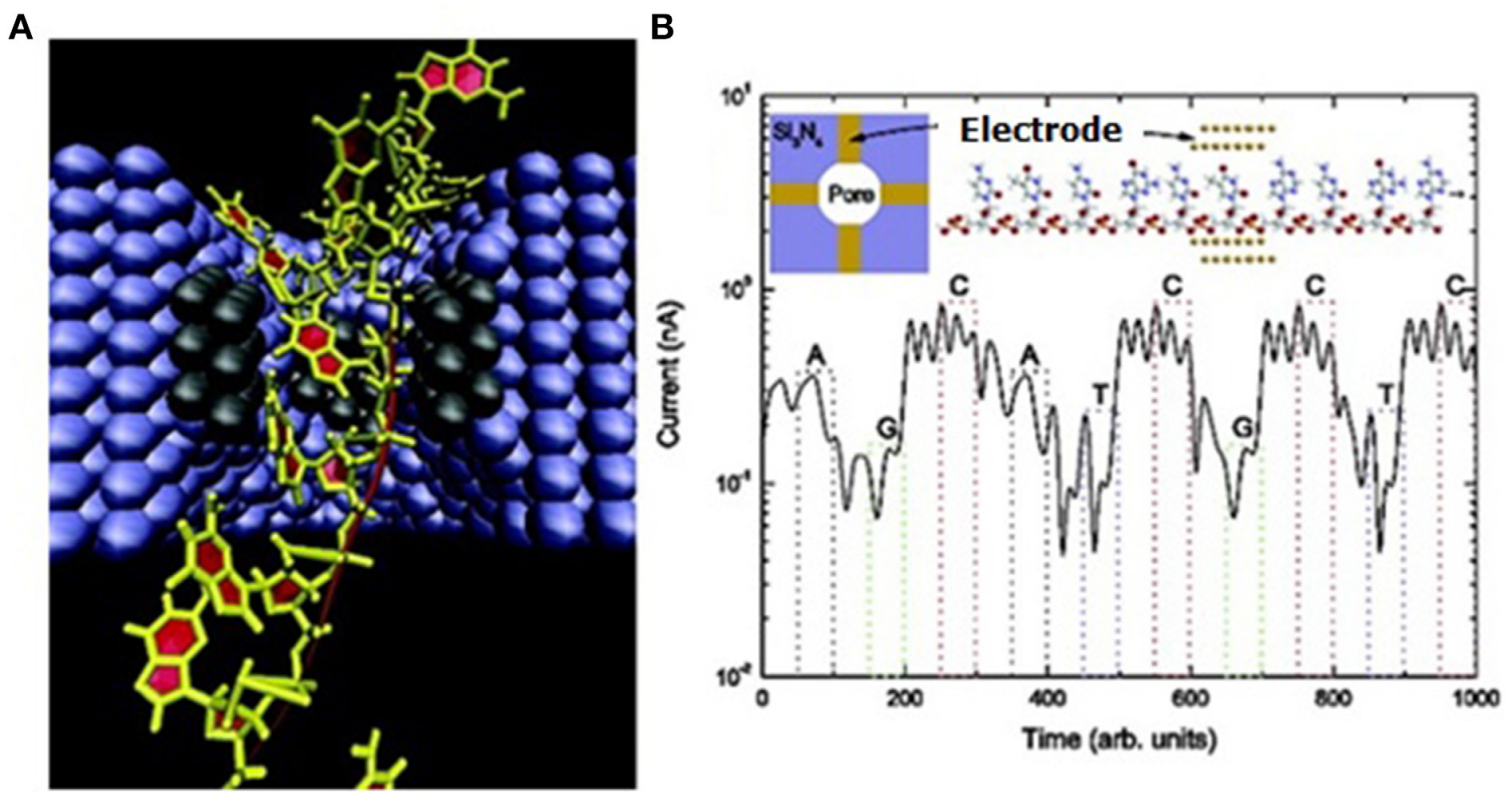
**Schematic illustration of SBET**. **(A)** Illustration of ssDNA traveling between a pair of tunneling electrodes in a nanochannel. **(B)** The upper left inset shows top view of the pore cross section embedded with two pairs of electrodes (gold rectangles). The upper right inset illustrates ssDNA and a pair of gold electrodes at atomic level. Lower shows the simulation result of a tunneling diagram of the ssDNA (sequence: AGCATCGCTC) (Lagerqvist et al., 2006). Reproduced by copyright permission of American Chemical Society.

In 2003, Lee and Thundat disclosed a nanoelectrode-gated tunneling method for DNA sequencing (Lee and Thundat, 2003). The hypothesis is based on the principle that each base has its distinct structure, and has specific perturbation effect on tunneling signals when each base is translocating between a pair of nanoelectrode tips (gate) perpendicular to DNA backbone. Lee et al. theoretically showed that the four bases have significant charge conductance which could possibly be detected when they are passing through a 1.5-nm gap between the nanoelectrodes (Lee, 2007). Theoretical calculation by di Ventra's group also supported this concept (Zwolak and Di Ventra, 2005). Their theoretical analysis showed that sequencing speed could reach up to 10^6^–10^7^ bases/s (Lee and Thundat, 2003; Lagerqvist et al., 2006). It has attracted increasing number of groups to develop new technologies to fabricate such devices. One example is shown in Figure 8.

However, the daunting challenges to fabricating such devices are: (1) to control the gap distance between the electrodes within 4 nm for recognition tunneling (Lindsay et al., 2010), (2) to obtain tip sharpness at the nanometer or even Angstrom scale for SBR, and (3) to operate such a device.

**Figure 9:**
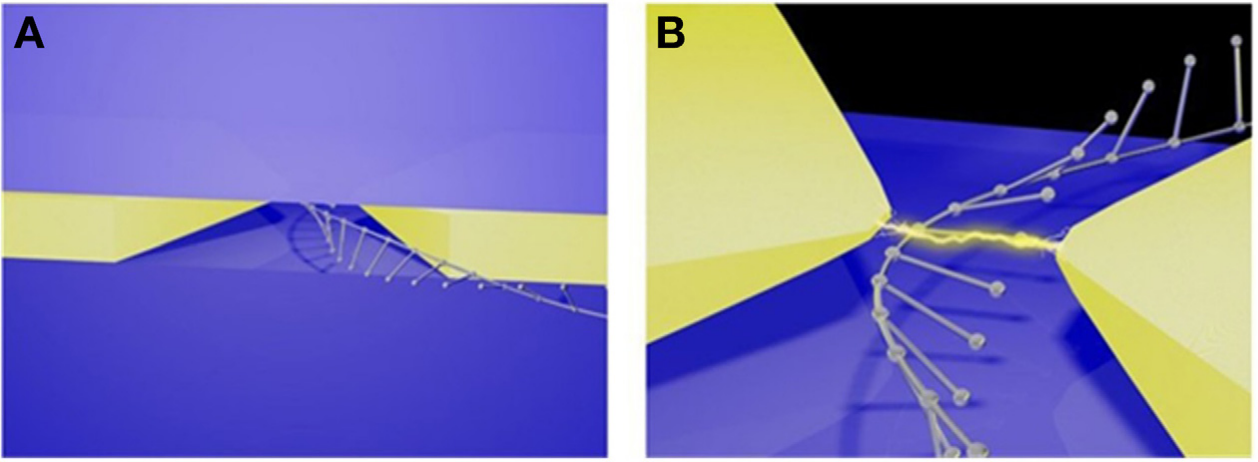
**Illustration of the SBET in a nanopore**. **(A)** An ssDNA molecule translocates through the electrode gap in-plane nanopore. **(B)** Continuous tunneling discrimination of individual bases of an ssDNA strand via the embedded electrodes (Tsutsui et al., 2011b). Reproduced by copyright permission of Nature Publishing Group.

***Direct tunneling***. In 2008, Collins and colleagues demonstrated that, using FEB, they embedded nanopores with 10-nm thick lateral chromium nanoelectrodes in a 2 to 50 nm spacing range (Gierhart et al., 2008). In the meantime, Kawai, Taniguchi and colleagues reported that gold electrode spacing could be controlled in a range from 0.5 to about 10 nm using the mechanically controllable break-junction method (MCBJ) (Tsutsui et al., 2008, 2009), initially developed by Reed, Tour and colleagues (Muller et al., 1996; Reed et al., 1997). These findings support the feasibility of SBET technology (Tanaka and Kawai, 2009). Later, they showed successful fabrication of 1-nm gapped Au electrode, which could statistically distinguish three bases except adenosine (Tsutsui et al., 2010). Again, with 1-nm gapped electrodes immersed in pure nucleotide solution, they also demonstrated that 5-mdC and 8-oxo-deoxyguanosine (8-oxo-dG) could be mathematically identified (Tsutsui et al., 2011a). To confine DNA strands within nano-sized space, they further embedded nanoelectrodes into an in-plane 15-nm SBN (Tsutsui et al., 2011b) (Figure 9), and sequenced short ssDNA and RNA fragments (Ohshiro et al., 2012), demonstrating the feasibility of SBET. Their sequence alignment using reference tunneling signatures of a series of oligomers implies their future direction of direct SBR. In 2013, Taniguchi cofounded Quantum Biosystems based on this technology (Karow, 2014b).

Although Taniguchi et al. have integrated gold electrodes with SBN, the nanopore size is too large to confine ssDNA molecules. A preferable pore diameter should be less than 4 nm (Lindsay et al., 2010). However, there are a number of challenges to embed metal electrodes into nanopores at the nanometer scale (Healy et al., 2012; Yokota et al., 2014). Some groups have therefore tried nonmetal compound materials.

In 2010, Postma proposed to sequence DNA within graphene nanogaps (Postma, 2010) using the nonlinear current-voltage characteristic of graphene electrode for base identification. Theoretical analysis suggested that sequencing accuracy could reach up to 100% when the gap-width was 1.6 nm. In 2011, calculation performed by Scheicher and colleagues also supported that it is possible to sequence DNA by measurements of tunneling current between graphene nanogap or electrodes (Prasongkit et al., 2011). They further proposed to modify graphene electrode edges by hydrogenation. MD simulation showed that edge-hydrogenated graphene electrodes would enhance conductance by about 3 orders of magnitude through interaction of hydrogen bonds with DNA bases (He et al., 2011) (see Section Single Base Resolution by Graphene and Other Solid-State Nanopores).

In 2010, Collin's group experimentally embedded carbon nanowire, gold, and chromium electrodes in a nanopore (Spinney et al., 2009; Sutanto et al., 2010). After assessing the device, they concluded that this sensor could be arrayed for parallel sequencing but fabrication of nanopores and electrodes at 1 nm scale may be necessary. In 2012, their detection of ss- and dsDNA translocation through the carbon electrode gap revealed that the device is sensitive (Spinney et al., 2012), but signals for base identification are not acquired due to large 3 nm nanopore and electrodes. Besides, Stein's group demonstrated that SWCNTs could be embedded in a nanopore (Jiang et al., 2010). Golovchenko, Branton, and colleagues reported an *in situ* growth method to embed SWCNTs across nanopores (Sadki et al., 2011). These provide new alternative approaches to minimize electrode size as diameter of the thinnest SWCNT is only 0.3 nm (Zhao et al., 2004).

**Figure 10:**
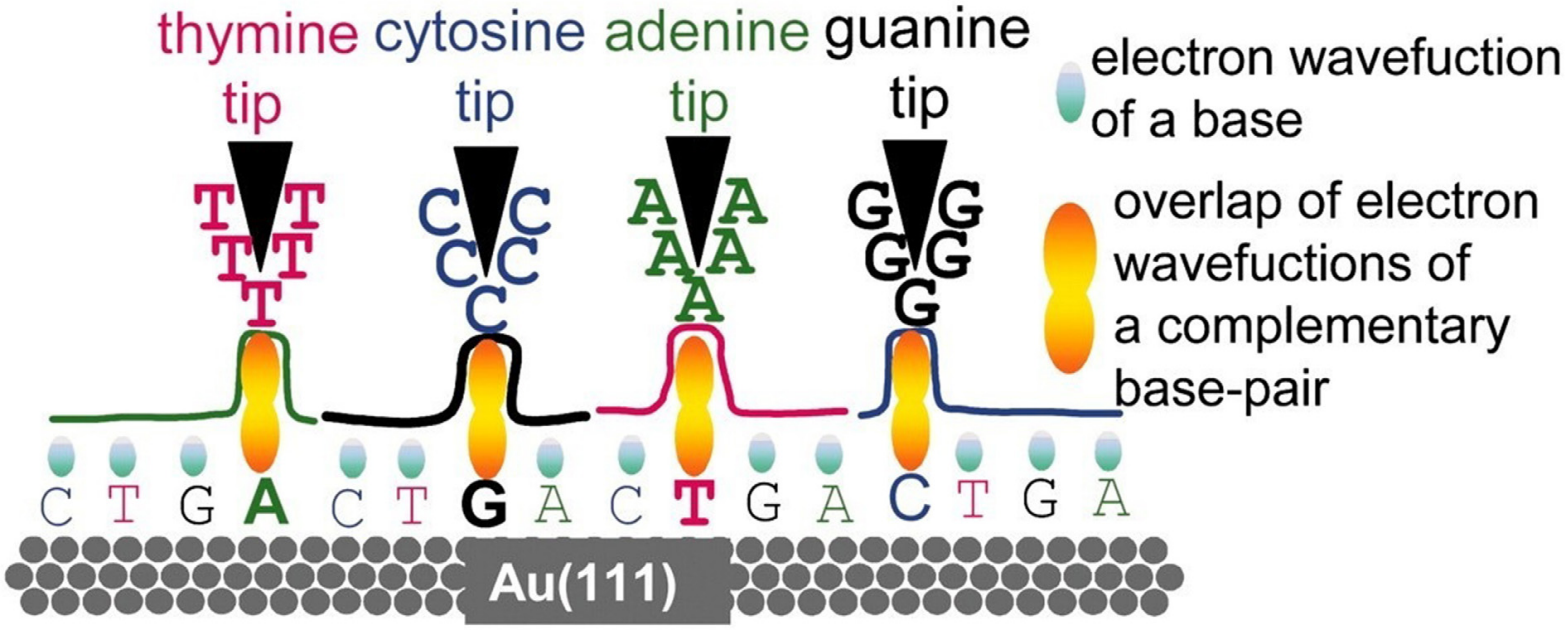
**Nucleotide-pairing based electron tunneling detection (Ohshiro and Umezawa, 2006)**. Reproduced by copyright permission of National Academy of Sciences.

***Hydrogen bond-mediated tunneling***. In 2005, Xu and colleagues reported their preliminary studies on DNA tunneling between Au(111) surface and tip of scanning tunneling microscope (STM), and presumed that these tunneling effect could be mediated by base pairing (Xu et al., 2005). Later, they reported (Xu et al., 2007) that the four DNA nucleosides and methylated bases deposited on Au(111) surface have specific tunneling conductance despite signal overlaps between bases are observed. This work demonstrated that it was possible to sequence DNA using electronic means. In 2006, Umezawa and colleagues reported that tips of STM with thiol derivatives of the 4 bases enhance electron tunneling signals when they are used to detect their complementary counterparts compared with their non-complementary ones (Figure 10), indicating that hydrogen bonds facilitate tunneling currents (Ohshiro and Umezawa, 2006).

**Figure 11:**
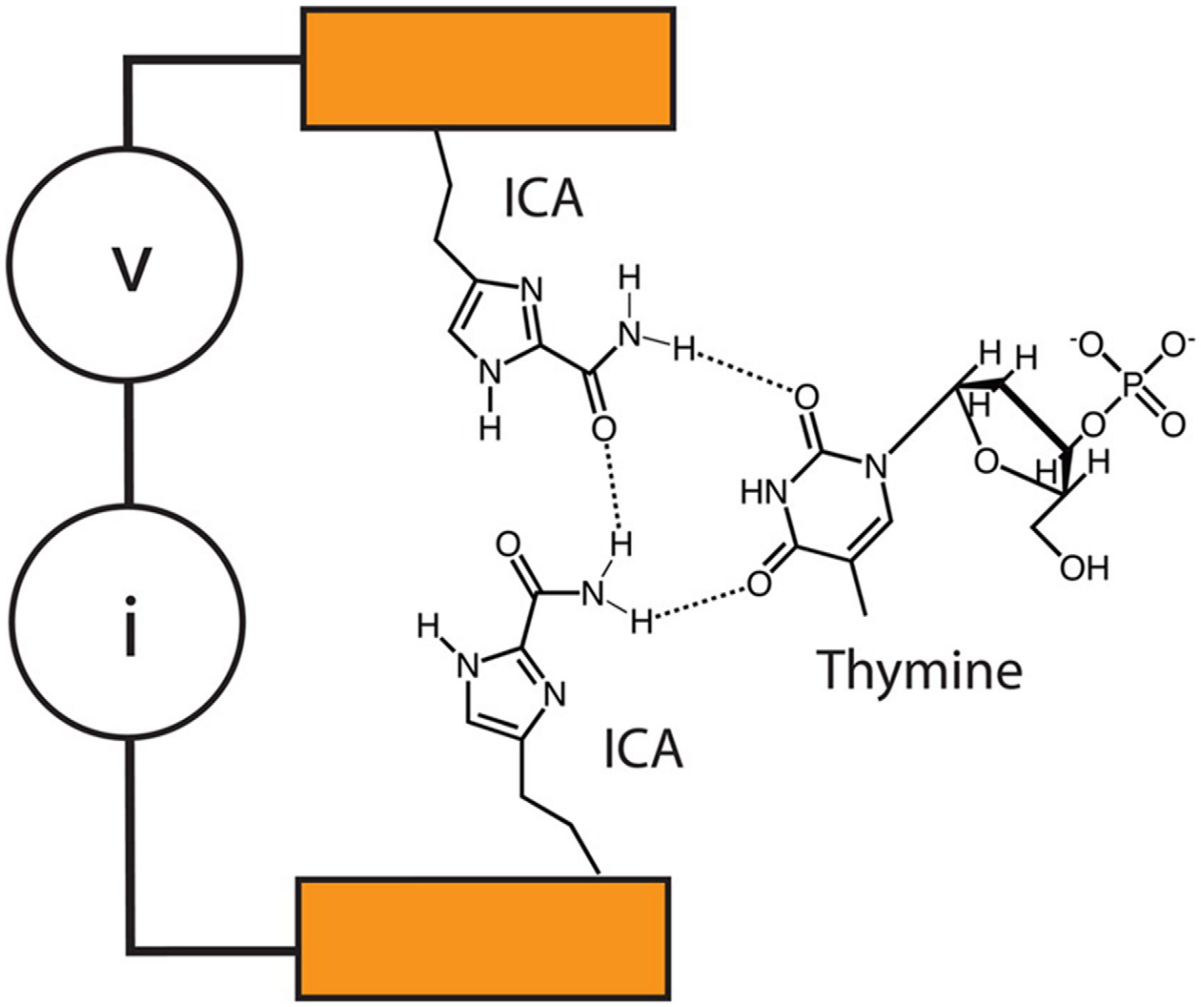
**Recognition tunneling sequencing**. Tunneling circuit is realized through the hydrogen bonds between ICA-functionalized tips and different DNA bases (Krishnakumar et al., 2013). Reproduced by copyright permission of American Chemical Society.

Based on tunneling enhancement by base paring, Lindsay's group proposed a sequencing concept called “Sequencing by Recognition (He et al., 2007)” (Figure 11), which relied on hydrogen bond-facilitated tunneling. According to Lindsay et al. 4 electrodes, each of which is coated with one of four molecule types complementary to A, C, G, and T, respectively, are used to identify their complementary counterparts. Immobilization of target DNA on surface and tunneling detection with tips of STM are complicated and time-consuming. However, it remains a promising platform for DNA sequencing if this tunneling detection could be combined with nanopores (Branton et al., 2008).

Lindsay's group previously showed that enhanced tunneling could be detected between complementary tip-base pairs, but not between non-complementary ones (He et al., 2007, 2009; Huang et al., 2010a). After their proof-of-principle work on tunneling identification of individual nucleobases (Chang et al., 2010), they tested base recognition tunneling, including methyl-cytosine, in different pentamers on Au(111) surface (Huang et al., 2010b). The results showed that functionalized tips are capable of identifying a base surrounded by other ones, although many factors, e.g., base stacking and tip sharpness, should be taken into consideration for more precise recognition. They further moved on to detect base tunneling within controlled nanogaps between electrode tips at the Angstrom precision by using piezoelectric transducer (Chang et al., 2011), which is an important step to combine tunneling detection with nanopore confinement of DNA molecules. Most notably, switching of the number of base-complementary molecules, which are used to functionalize electrode tips, from 4 to 1 by using a universal recognition molecule, 4(5)-substituted imidazole-2-carboxamide (ICA), is considered as a quantum leap (Liang et al., 2012). This could enable a single instead of four pairs of electrodes to detect all the four bases, and circumvent challenges in embedding electrodes into nanopore and subsequent signal analysis. Recently, this group has reported that palladium electrodes functionalized with an ICA compound could detect not only tunneling signals of the 4 normal bases, but also 5-mdC (Chang et al., 2012), though acquisition of unambiguously distinguishable tunneling signals across bases needs further improvements. The double-functionalized gold electrodes proposed by Scheicher and colleagues (Pathak et al., 2012; Prasongkit et al., 2013) and titanium nitride electrodes functionalized with a thiol-bearing compound proposed by Chen (2013) could be useful for improving base recognition. Moreover, ICA-coated electrodes could slow DNA translocation speed through nanopore down to about 200 μs/base under the applied voltage between 60 and 80 mV (Krishnakumar et al., 2013). Strikingly, ICA-coated electrodes are potential to detect peptides (Zhao et al., 2014), probably opening up the door to single-molecule proteomics if the amplitude range of tunneling is wide enough to distinguish at least 20 animo acids.

#### Measurement of transverse conductance of DNA bases

Based on density functional theory, Prezhdo's, Xu's, and Nikolić's groups (Nelson et al., 2010; Ouyang et al., 2011; Saha et al., 2012) independently proposed nanopore-related sequencing methods. Briefly, when a base is inserted into a nanopore in a metallic monolayer graphene nanoribbon, it will modulate edge conductance currents. These conductance is base-specific and can be enhanced in the micro- to milliampere range, compared with the picoampere scale of ionic current. Therefore, an ssDNA chain can be sequenced when it passes through the nanopore. Later, Lambert and colleagues (Sadeghi et al., 2014) further introduced this sequencing method by using nanopores in bilayer graphene. Theoretical calculation indicates that electrical current signals have a high signal-to-noise ratio compared with conventional ionic current measurement. The authors also predicted that it could be integrated with CMOS technology. Balatsky and coworkers (Ahmed et al., 2014) also proposed multilayered graphene-based nanopore sequencing device combined with “multi-point cross-correlation (MPCC)” method. In this design, transverse current across each graphene layer is independently recorded during DNA translocation through the pores. Then the signals between each nanopore are subject to MPCC, which could theoretically enhance signal-to-noise ratio.

#### Concurrent detection of ionic current blockage and other signals

In 2011, Albrecht, Edel, and coworkers presented an idea to combine sequencing by detection of ionic current blockage and tunneling current (Ivanov et al., 2011). They embedded Pt electrodes in a SBN, and simultaneously measured tunneling and ionic currents. Proof-of-principle tests showed that events of DNA translocation through nanopore could be concurrently detected, indicating that this concept is possible. Then, this group developed a method called “electron-beam-induced deposition (EBID),” an effective way to precisely control electrodes spacing (Ivanov et al., 2014). Furthermore, EBID is also suitable for fabrication of nanopore arrays. In 2013, Radenovic's group reported a device by combining fabrication of nanopores in graphene nanoribbon with semiconductor technology, and demonstrated that it could simultaneously detect ionic current blockage and electronic conductance (see Section Field Effect Transistor) (Traversi et al., 2013). Recently, Forró, Radenovic, and colleagues reported electron beam lithography (EBL) fabrication of sub-10 nm nanogap electrodes and concurrent detection of ionic current blockage and tunneling (Fanget et al., 2014).

In 2014, Lal's group reported a designed nanopore device that can simultaneously perform AFM imaging and ionic current detection, suggesting that it could be used for DNA sequencing (Connelly et al., 2014). Later, Timp's group reported concurrent measurements of ionic current and force topography while threading individual ssDNA strands through SBNs (Nelson et al., 2014). An important phenomenon they found is coexistence of frictionless sliding and “slip-stick” motions, which could become error sources during sequencing. Minimizing the latter, through surface passivation as an example, could enhance sequencing precision. Signal analysis supports that SBR is possible using nanopores with diameters no more than 1.5 nm. Early, Wang, Liang, and colleagues proposed that individual bases, including 5-mdC, could be recognized through detection of force topographies by pulling DNA strands through ~1.0-nm axisymmetric graphene pores by AFM or optical tweezers (Zhang et al., 2014).

#### Field effect transistor

**Figure 12:**
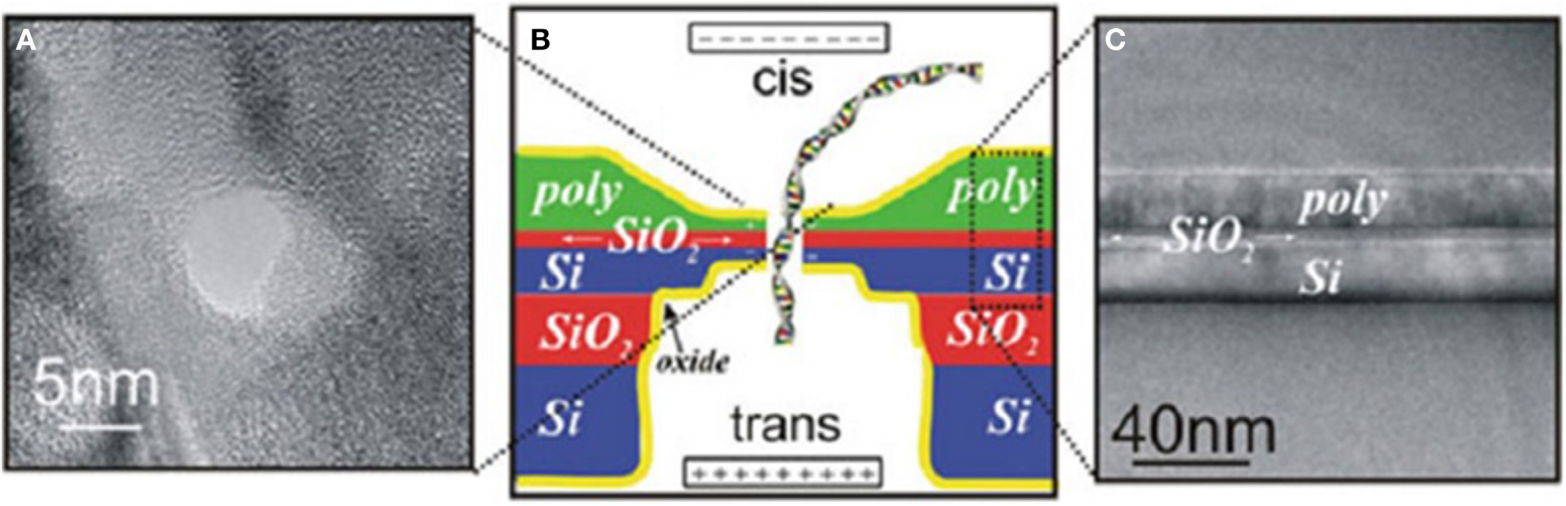
**Nanopore capacitor**. **(A)** A TEM top-view of the nanopore capacitor device. **(B)** Schematic illustration of the nanopore capacitor. **(C)** A TEM side-view of the nanopore capacitor (Gracheva et al., 2006). Reproduced by copyright permission of IOP Publishing Ltd.

In 2005, Heng et al. proposed a design that used nanopore capacitor made in a metal-oxide-semiconductor (MOS) membrane to sense bases when an ssDNA strand is translocating through the pore (Heng et al., 2005). Their simulation results demonstrated that such a device holds to sequence DNA (Heng et al., 2005; Gracheva et al., 2006; Sigalov et al., 2008) (Figure 12). In 2010, Leroux presented a similar design that used a nanopore in a semiconductor-oxide-semiconductor membrane to distinguish individual bases (Leroux et al., 2010). Their theoretical analysis showed that this is possible. In 2012, Yan's group proposed a sequencing method by using single-electron transistor-based nanopore (Guo et al., 2012). Based on simulation results, they concluded that bases could be identified only in some regions. Although supporting evidence from experiments are currently unavailable, these transistor-based approaches can potentially be integrated with semiconductor technology for massive parallelization.

In 2009, Kim, Rossnagel, and coworkers demonstrated fabrication of array of ionic field effect transistor (IFET) nanopores by combining EBL and atomic layer deposition (Nam et al., 2009). Recently, Lieber's group has reported fabrication of FET nanopores via FEB drilling through both the edge of a silicon nanowire and its silicon nitride support membrane (Xie et al., 2012). Their experiments on DNA translocation through these FET nanopores showed that the amplitude of FET signals is about ten-fold larger than that of ionic current counterparts, and have higher signal-to-noise ratio, implying higher bandwidth detection and accuracy, respectively. Data analysis supported that FET signals in this transistor follow the mechanism of local potential change-related sensing rather than that of charge-based sensing. The authors predicted that the nanowire could be replaced by monolayer graphene or integrated with biologically engineered protein pores for SBR.

**Figure 13:**
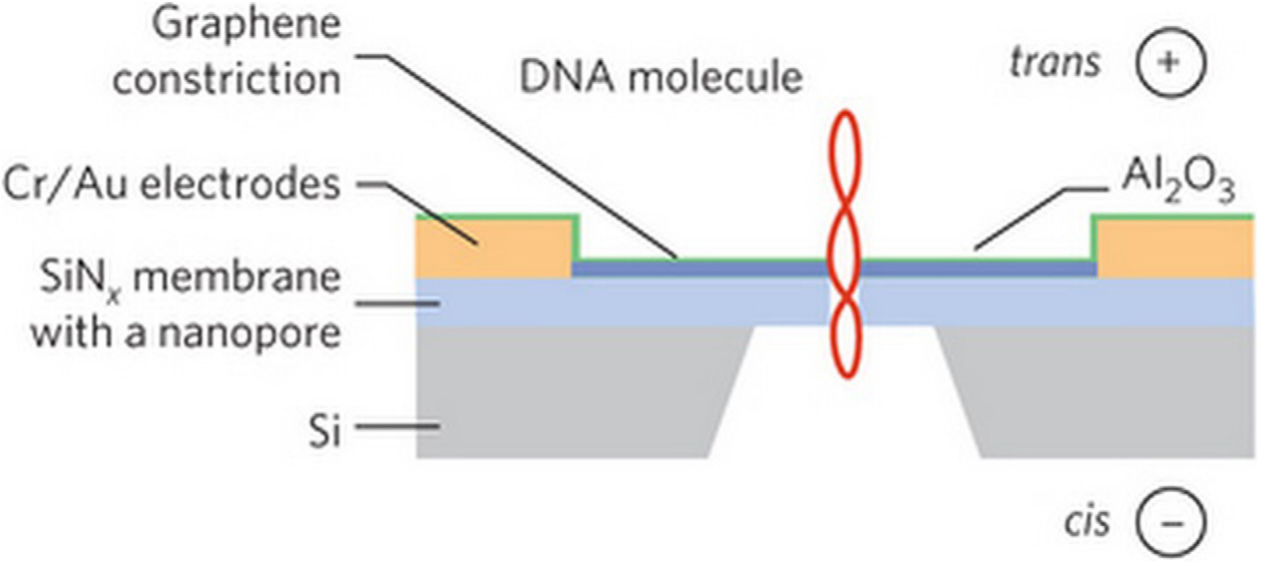
**Schematic illustration of the fabrication of an FET nanopore, in which a dsDNA is translocating through the pore (not to scale) (Traversi et al., 2013)**. Reproduced by copyright permission of Nature Publishing Group.

In 2013, Drndić's group described fabrication of FET monolayer graphene nanopores with diameters between 2 and 10 nm using EBL (Puster et al., 2013). Radenovic's group reported fabrication of graphene-based FET sensors and signal tests on DNA translocation (Traversi et al., 2013) (Figure 13). Their results also support local potential change-related sensing mechanism. Leburton proposed a four-layer FET nanopore sequencing design called graphene quantum point contact device (Girdhar et al., 2013), where the top graphene layer controlls DNA translocation speed, the second confines lateral positioning of the bases, the third detectes lateral conductance, and the bottom alteres the carrier concentration. These compelling works suggest that further innovation toward sequencing by FET sensors will be soon emerging.

It should be noticed that dsDNA is used to illustrate sequencing principle in Figures 12, 13. The devices could use ssDNA for real sequencing.

## The future of nanopore sequencing

Nanopore sequencing technologies have been progressing at an unprecedentedly fast speed whereas the 2nd-gen sequencers have been paving the way for sequencing-based personalized medicine. It can be envisioned that novel sequencing concepts and technologies, including methods/materials for nanopore fabrication, parallelization techniques for nanopore arrays and detection, and speed control of DNA translocation, etc., will facilitate the development of directly-reading nanopore sequencers that presently rely on indirect reading. It can also be foreseen that the ultimate goal of clinical sequencing will be achieved in the near future along with the evolution in sequencing technology and sequence annotation.

Besides the four gold standards, novel nanopore-based sequencers are exhibiting their potential to detect cytosine methylation (Clarke et al., 2009; Tsutsui et al., 2011a; Laszlo et al., 2013; Schreiber et al., 2013). In addition, nanopore can identify 5-hydroxymethyl-2′-deoxycytosine (5-hmdC) (Wallace et al., 2010; Laszlo et al., 2013; Li et al., 2013b), 8-oxo-dG (Schibel et al., 2010; Tsutsui et al., 2011a) and abasic sites (An et al., 2012a,b; Kim et al., 2013b; Marshall et al., 2014). Based-on MD simulations, Wanunu, Drndić and coworkers reported that SBN is able to distinguish 5-mdC from 5-hmdC by sequencing a few hundred molecules (Wanunu et al., 2010a). It is therefore necessary to redefine sequencing accuracy to include not only 4 normal bases but also their modified/damaged derivatives, many of which are involved in diseases (Korlach and Turner, 2012; Vogelstein et al., 2013). However, it remains a huge challenge of how to harness nanopore-based sequencing methods to directly distinguish all of alterations beyond mentioned above. Thus, the revised four gold standards would be: (1) high accuracy (no more than one error in every 10,000 bases including normal bases and their modified forms), (2) long read length (10 of Kb or longer), (3) high throughput and short TTR (in the matter of hours or even minutes), and (4) low cost (much less than $1000/genome). These standards are not only applicable to DNA sequencing, but also to direct RNA sequencing (Ayub and Bayley, 2012; Ayub et al., 2013).

The concept of “$1000 Genome” is by no means the clinical cost. Data interpretation is costly and time-consuming. This is currently becoming a bottle neck next to sequencing itself. On the other hand, cancer patients may need multiple sequencing to monitor genome stability during treatment. Even if cost for each sequencing and interpretation is $1000, it is still expensive for ordinary families. Therefore, how to popularize sequencing-based personalized medicine will require joint efforts from multiple fronts.

### Conflict of interest statement

The authors declare that the research was conducted in the absence of any commercial or financial relationships that could be construed as a potential conflict of interest.

## References

[B1] A Genomeweb Staff Reporter (2013a). Illumina licenses nanopore-based sequencing technology from UAB-UW, in GenomeWeb. Available onlie at: http://www.genomeweb.com/sequencing/illumina-licenses-nanopore-based-sequencing-technology-uab-uw (Accessed July 26, 2014).

[B2] A Genomeweb Staff Reporter (2013b). Oxford Nanopore to launch early-access program for MinION sequencer next month, in GenomeWeb. Available online at: http://www.genomeweb.com/sequencing/oxford-nanopore-launch-early-access-program-minion-sequencer-next-month (Accessed July 26, 2014).

[B3] A Genomeweb Staff Reporter (2013c). Roche shutting down 454 sequencing business, in GenomeWeb. Available online at: http://www.genomeweb.com/sequencing/roche-shutting-down-454-sequencing-business (Accessed July 26, 2014).

[B4] Ahmed T. Haraldsen J. Rehr J. J. Di Ventra M. Schuller I. Balatsky A. V. (2014). Correlation dynamics and enhanced signals for the identification of serial biomolecules and DNA bases. Nanotechnology 25, 125705. 10.1088/0957-4484/25/12/125705 24577191

[B5] Akeson M. Branton D. Kasianowicz J. J. Brandin E. Deamer D. W. (1999). Microsecond time-scale discrimination among polycytidylic acid, polyadenylic acid, and polyuridylic acid as homopolymers or as segments within single RNA molecules. Biophys. J. 77, 3227–3233. 10.1016/S0006-3495(99)77153-5 10585944 PMC1300593

[B6] An N. Fleming A. M. White H. S. Burrows C. J. (2012a). Crown ether-electrolyte interactions permit nanopore detection of individual DNA abasic sites in single molecules. Proc. Natl. Acad. Sci. U.S.A. 109, 11504–11509. 10.1073/pnas.1201669109 22711805 PMC3406832

[B7] An N. White H. S. Burrows C. J. (2012b). Modulation of the current signatures of DNA abasic site adducts in the α-hemolysin ion channel. Chem. Comm. 48, 11410–11412. 10.1039/C2CC36366F 23076012 PMC3738171

[B8] Anderson A. (2012). Columbia team pursues integrated electronic approaches for measuring multiplexed nanopore signals, in In Sequence/GenomeWeb. Available online at: http://www.genomeweb.com/sequencing/columbia-team-pursues-integrated-electronic-approaches-measuring-multiplexed-nan (Accessed July 30, 2014).

[B9] Astier Y. Braha O. Bayley H. (2006). Toward single molecule DNA sequencing: direct identification of ribonucleoside and deoxyribonucleoside 5′-monophosphates by using an engineered protein nanopore equipped with a molecular adapter. J. Am. Chem. Soc. 128, 1705–1710. 10.1021/ja057123+ 16448145

[B10] Ayub M. Bayley H. (2012). Individual RNA base recognition in immobilized oligonucleotides using a protein nanopore. Nano Lett. 12, 5637–5643. 10.1021/nl3027873 23043363 PMC3505278

[B11] Ayub M. Hardwick S. W. Luisi B. F. Bayley H. (2013). Nanopore-based identification of individual nucleotides for direct RNA sequencing. Nano Lett. 13, 6144–6150. 10.1021/nl403469r 24171554 PMC3899427

[B12] Baaken G. Ankri N. Schuler A.-K. Rühe J. Behrends J. C. (2011). Nanopore-based single-molecule mass spectrometry on a lipid membrane microarray. ACS Nano 5, 8080–8088. 10.1021/nn202670z 21932787

[B13] Bae S. Kim H. Lee Y. Xu X. Park J.-S. Zheng Y.. (2010). Roll-to-roll production of 30-inch graphene films for transparent electrodes. Nat. Nanotechnol. 5, 574–578. 10.1038/nnano.2010.132 20562870

[B14] Bai J. Wang D. Nam S.-W. Peng H. Bruce R. Gignac L.. (2014). Fabrication of sub-20 nm nanopore arrays in membranes with embedded metal electrodes at wafer scales. Nanoscale 6, 8900–8906. 10.1039/C3NR06723H 24964839

[B15] Bailo E. Deckert V. (2008). Tip-enhanced Raman spectroscopy of single RNA strands: towards a novel direct-sequencing method. Angew. Chem. Int. Ed. 47, 1658–1661. 10.1002/anie.200704054 18188855

[B16] Balagurusamy V. S. Weinger P. Ling X. S. (2010). Detection of DNA hybridizations using solid-state nanopores. Nanotechnology 21, 335102. 10.1088/0957-4484/21/33/335102 20657045 PMC4811674

[B17] Bayley H. (2006). Sequencing single molecules of DNA. Curr. Opin. Chem. Biol. 10, 628–637. 10.1016/j.cbpa.2006.10.040 17113816

[B18] Bayley H. (2010). Nanotechnology: holes with an edge. Nature 467, 164–165. 10.1038/467164a 20829786

[B19] Bell D. C. Thomas W. K. Murtagh K. M. Dionne C. A. Graham A. C. Anderson J. E.. (2012). DNA base identification by electron microscopy. Microsc. Microanal. 18, 1049–1053. 10.1017/S1431927612012615 23046798

[B20] Benner S. Chen R. J. A. Wilson N. A. Abu-Shumays R. Hurt N. Lieberman K. R.. (2007). Sequence-specific detection of individual DNA polymerase complexes in real time using a nanopore. Nat. Nanotechnol. 2, 718–724. 10.1038/nnano.2007.344 18654412 PMC2507869

[B21] Blanco L. Bernad A. Lázaro J. M. Martín G. Garmendia C. Salas M. (1989). Highly efficient DNA synthesis by the phage phi 29 DNA polymerase. Symmetrical mode of DNA replication. J. Biol. Chem. 264, 8935–8940. 2498321

[B22] Branton D. Deamer D. W. Marziali A. Bayley H. Benner S. A. Butler T.. (2008). The potential and challenges of nanopore sequencing. Nat. Biotechnol. 26, 1146–1153. 10.1038/nbt.1495 18846088 PMC2683588

[B23] Butler T. Z. Pavlenok M. Derrington I. M. Niederweis M. Gundlach J. H. (2008). Single-molecule DNA detection with an engineered MspA protein nanopore. Proc. Natl. Acad. Sci. U.S.A. 105, 20647–20652. 10.1073/pnas.0807514106 19098105 PMC2634888

[B24] Cai Q. Ledden B. Krueger E. Golovchenko J. A. Li J. (2006). Nanopore sculpting with noble gas ions. J. Appl. Phys. 100, 024914. 10.1063/1.2216880 21331305 PMC3039599

[B25] Chang S. He J. Zhang P. Gyarfas B. Lindsay S. (2011). Gap distance and interactions in a molecular tunnel junction. J. Am. Chem. Soc. 133, 14267–14269. 10.1021/ja2067737 21838292 PMC3367717

[B26] Chang S. Huang S. He J. Liang F. Zhang P. Li S.. (2010). Electronic signatures of all four DNA nucleosides in a tunneling gap. Nano Lett. 10, 1070–1075. 10.1021/nl1001185 20141183 PMC2836180

[B27] Chang S. Sen S. Zhang P. Gyarfas B. Ashcroft B. Lefkowitz S.. (2012). Palladium electrodes for molecular tunnel junctions. Nanotechnology 23, 425202. 10.1088/0957-4484/23/42/425202 23037952 PMC3501205

[B28] Chansin G. A., T. Mulero R. Hong J. Kim M. J. Demello A. J. Edel J. B. (2007). Single-molecule spectroscopy using nanoporous membranes. Nano Lett. 7, 2901–2906. 10.1021/nl071855d 17718589

[B29] Chen P. Mitsui T. Farmer D. B. Golovchenko J. Gordon R. G. Branton D. (2004). Atomic layer deposition to fine-tune the surface properties and diameters of fabricated nanopores. Nano Lett. 4, 1333–1337. 10.1021/nl0494001 24991194 PMC4076156

[B30] Chen X. (2013). DNA sequencing with titanium nitride electrodes. Int. J. Quantum Chem. 113, 2295–2305 10.1002/qua.24451

[B31] Cheng P. Oliver P. M. Barrett M. J. Vezenov D. (2012). Progress toward the application of molecular force spectroscopy to DNA sequencing. Electrophoresis 33, 3497–3505. 10.1002/elps.201200351 23161379 PMC3815542

[B32] Cherf G. M. Lieberman K. R. Rashid H. Lam C. E. Karplus K. Akeson M. (2012). Automated forward and reverse ratcheting of DNA in a nanopore at 5-Å precision. Nat. Biotechnol. 30, 344–348. 10.1038/nbt.2147 22334048 PMC3408072

[B33] Chu J. González-López M. Cockroft S. L. Amorin M. Ghadiri M. R. (2010). Real-time monitoring of DNA polymerase function and stepwise single-nucleotide DNA strand translocation through a protein nanopore. Angew. Chem. Int. Ed. 49, 10106–10109. 10.1002/anie.201005460 21105031 PMC3132071

[B34] Church G. Deamer D. Branton D. Baldarelli R. Kasianowicz J. (1998). Characterization of Individual Polymer Molecules Based on Monomer-Interface Interactions. US Patent No. 5795782, Brookline; Santa Cruz; Lexington; Natick; Darnestown.

[B35] Clamer M. Höfler L. Mikhailova E. Viero G. Bayley H. (2013). Detection of 3′-end RNA uridylation with a protein nanopore. ACS Nano 8, 1364–1374. 10.1021/nn4050479 24369707 PMC3936189

[B36] Clarke J. Wu H.-C. Jayasinghe L. Patel A. Reid S. Bayley H. (2009). Continuous base identification for single-molecule nanopore DNA sequencing. Nat. Nanotechnol. 4, 265–270. 10.1038/nnano.2009.12 19350039

[B37] Cockroft S. L. Chu J. Amorin M. Ghadiri M. R. (2008). A single-molecule nanopore device detects DNA polymerase activity with single-nucleotide resolution. J. Am. Chem. Soc. 130, 818–820. 10.1021/ja077082c 18166054 PMC2453067

[B38] Connelly L. S. Meckes B. Larkin J. Gillman A. L. Wanunu M. Lal R. (2014). Graphene nanopore support system for simultaneous high-resolution AFM imaging and conductance measurements. ACS Appl. Mater. Interfaces 6, 5290–5296. 10.1021/am500639q 24581087 PMC4232248

[B39] Cracknell J. A. Japrung D. Bayley H. (2013). Translocating kilobase RNA through the staphylococcal α-hemolysin nanopore. Nano Lett. 13, 2500–2505. 10.1021/nl400560r 23678965 PMC3712197

[B40] Davies K. (2012). Oxford strikes first in DNA sequencing nanopore wars, in Bio-IT World. Available online at: http://www.bio-itworld.com/news/02/17/12/Oxford-strikes-first-in-DNA-sequencing-nanopore-wars.html (Accessed July 8, 2014).

[B41] Deamer D. W. Akeson M. (2000). Nanopores and nucleic acids: prospects for ultrarapid sequencing. Trends Biotech. 18, 147–151. 10.1016/S0167-7799(00)01426-8 10740260

[B42] Deamer D. W. Branton D. (2002). Characterization of nucleic acids by nanopore analysis. Acc. Chem. Res. 35, 817–825. 10.1021/ar000138m 12379134

[B43] Dekker C. (2007). Solid-state nanopores. Nat. Nanotechnol. 2, 209–215 10.1038/nnano.2007.27 18654264

[B44] Derrington I. M. Butler T. Z. Collins M. D. Manrao E. Pavlenok M. Niederweis M.. (2010). Nanopore DNA sequencing with MspA. Proc. Natl. Acad. Sci. U.S.A. 107, 16060–16065. 10.1073/pnas.1001831107 20798343 PMC2941267

[B45] Ding F. Manosas M. Spiering M. M. Benkovic S. J. Bensimon D. Allemand J.-F.. (2012). Single-molecule mechanical identification and sequencing. Nat. Meth. 9, 367–372. 10.1038/nmeth.1925 22406857 PMC3528176

[B46] Eid J. Fehr A. Gray J. Luong K. Lyle J. Otto G.. (2009). Real-time DNA sequencing from single polymerase molecules. Science 323, 133–138. 10.1126/science.1162986 19023044

[B47] Erlich Y. Mitra P. P. Delabastide M. McCombie W. R. Hannon G. J. (2008). Alta-Cyclic: a self-optimizing base caller for next-generation sequencing. Nat. Meth. 5, 679–682. 10.1038/nmeth.1230 18604217 PMC2978646

[B48] Ervin E. N. Barrall G. A. Pal P. Bean M. K. Schibel A. P. Hibbs A. D. (2014). Creating a single sensing zone within an alpha-hemolysin pore via site-directed mutagenesis. BioNanoScience 4, 78–84. 10.1007/s12668-013-0119-0 24678449 PMC3963172

[B49] Faller M. Niederweis M. Schulz G. E. (2004). The structure of a mycobacterial outer-membrane channel. Science 303, 1189–1192. 10.1126/science.1094114 14976314

[B50] Fanget A. Traversi F. Khlybov S. Granjon P. Magrez A. Forro L.. (2014). Nanopore integrated nanogaps for DNA detection. Nano Lett. 14, 244–249. 10.1021/nl403849g 24308689

[B51] Fischbein M. D. Drndić M. (2008). Electron beam nanosculpting of suspended graphene sheets. Appl. Phys. Lett. 93, 113107 10.1063/1.2980518

[B52] Fologea D. Uplinger J. Thomas B. McNabb D. S. Li J. (2005). Slowing DNA translocation in a solid-state nanopore. Nano Lett. 5, 1734–1737. 10.1021/Nl051063o 16159215 PMC3037730

[B53] Garaj S. Hubbard W. Reina A. Kong J. Branton D. Golovchenko J. (2010). Graphene as a subnanometre trans-electrode membrane. Nature 467, 190–193. 10.1038/Nature09379 20720538 PMC2956266

[B54] Garaj S. Liu S. Golovchenko J. A. Branton D. (2013). Molecule-hugging graphene nanopores. Proc. Natl. Acad. Sci. U.S.A. 110, 12192–12196. 10.1073/pnas.1220012110 23836648 PMC3725097

[B55] Gierhart B. C. Howitt D. G. Chen S. J. Zhu Z. Kotecki D. E. Smith R. L.. (2008). Nanopore with transverse nanoelectrodes for electrical characterization and sequencing of DNA. Sensor Actuat. B Chem. 132, 593–600. 10.1016/j.snb.2007.11.054 19584949 PMC2706128

[B56] Girdhar A. Sathe C. Schulten K. Leburton J. P. (2013). Graphene quantum point contact transistor for DNA sensing. Proc. Natl. Acad. Sci. U.S.A. 110, 16748–16753. 10.1073/pnas.1308885110 24082108 PMC3801026

[B57] Girit Ç. Ö. Meyer J. C. Erni R. Rossell M. D. Kisielowski C. Yang L.. (2009). Graphene at the edge: stability and dynamics. Science 323, 1705–1708. 10.1126/science.1166999 19325110

[B58] Gouaux J. E. Braha O. Hobaugh M. R. Song L. Cheley S. Shustak C.. (1994). Subunit stoichiometry of staphylococcal α-hemolysin in crystals and on membranes: a heptameric transmembrane pore. Proc. Natl. Acad. Sci. U.S.A. 91, 12828–12831. 10.1073/pnas.91.26.12828 7809129 PMC45533

[B59] Gracheva M. E. Xiong A. Aksimentiev A. Schulten K. Timp G. Leburton J.-P. (2006). Simulation of the electric response of DNA translocation through a semiconductor nanopore–capacitor. Nanotechnology 17, 622 10.1088/0957-4484/17/3/002

[B60] Greenleaf W. J. Block S. M. (2006). Single-molecule, motion-based DNA sequencing using RNA polymerase. Science 313, 801–801. 10.1126/science.1130105 16902131 PMC1865524

[B61] Guo J. Xu N. Li Z. Zhang S. Wu J. Kim D. H.. (2008). Four-color DNA sequencing with 3′-*O*-modified nucleotide reversible terminators and chemically cleavable fluorescent dideoxynucleotides. Proc. Natl. Acad. Sci. U.S.A. 105, 9145–9150. 10.1073/pnas.0804023105 18591653 PMC2442126

[B62] Guo Y.-D. Yan X.-H. Xiao Y. (2012). Computational investigation of DNA detection using single-electron transistor-based nanopore. J. Phys. Chem. C 116, 21609–21614 10.1021/jp305909p

[B63] Gyarfas B. Olasagasti F. Benner S. Garalde D. Lieberman K. R. Akeson M. (2009). Mapping the position of DNA polymerase-bound DNA templates in a nanopore at 5 Å resolution. ACS Nano 3, 1457–1466. 10.1021/nn900303g 19489560

[B64] Hall A. R. Scott A. Rotem D. Mehta K. K. Bayley H. Dekker C. (2010). Hybrid pore formation by directed insertion of α-haemolysin into solid-state nanopores. Nat. Nanotechnol. 5, 874–877. 10.1038/nnano.2010.237 21113160 PMC3137937

[B65] Hamburg M. A. Collins F. S. (2010). The path to personalized medicine. N. Eng. J. Med. 363, 301–304. 10.1056/NEJMp1006304 20551152

[B66] Harris T. D. Buzby P. R. Babcock H. Beer E. Bowers J. Braslavsky I.. (2008). Single-molecule DNA sequencing of a viral genome. Science 320, 106–109. 10.1126/science.1150427 18388294

[B67] He J. Lin L. Liu H. Zhang P. Lee M. Sankey O.. (2009). A hydrogen-bonded electron-tunneling circuit reads the base composition of unmodified DNA. Nanotechnology 20, 075102. 10.1088/0957-4484/20/7/075102 19417406 PMC2678007

[B68] He J. Lin L. Zhang P. Lindsay S. (2007). Identification of DNA basepairing via tunnel-current decay. Nano Lett. 7, 3854–3858. 10.1021/nl0726205 18041859 PMC2311509

[B69] He Y. Scheicher R. H. Grigoriev A. Ahuja R. Long S. Huo Z. (2011). Enhanced DNA sequencing performance through edge-hydrogenation of graphene electrodes. Adv. Func. Mat. 21, 2674–2679 10.1002/adfm.201002530

[B70] Healy K. Ray V. Willis L. J. Peterman N. Bartel J. Drndiæ M. (2012). Fabrication and characterization of nanopores with insulated transverse nanoelectrodes for DNA sensing in salt solution. Electrophoresis 33, 3488–3496. 10.1002/elps.201200350 23161707 PMC3828733

[B71] Heger M. (2014a). At AGBT, first data from Oxford Nanopore presented as company issues early access invites, in In Sequence/GenomeWeb. Available online at: http://www.genomeweb.com/sequencing/agbt-first-data-oxford-nanopore-presented-company-issues-early-access-invites (Accessed July 26, 2014).

[B72] Heger M. (2014b). Illumina launches two new NGS instruments: desktop platform and ‘factory-scale’ system, in In Sequence/GenomeWeb. Available online at: http://www.genomeweb.com/sequencing/illumina-launches-two-new-ngs-instruments-desktop-platform-and-factory-scale-sys (Accessed July 26, 2014).

[B73] Heng J. B. Aksimentiev A. Ho C. Dimitrov V. Sorsch T. W. Miner J. F.. (2005). Beyond the gene chip. Bell Labs Tech. J. 10, 5–22. 10.1002/bltj.20102 18815623 PMC2546600

[B74] Henrickson S. E. Misakian M. Robertson B. Kasianowicz J. J. (2000). Driven DNA transport into an asymmetric nanometer-scale pore. Phys. Rev. Lett. 85, 3057–3060. 10.1103/PhysRevLett.85.3057 11006002

[B75] Hong J. Lee Y. Chansin G. Edel J. B. Demello A. J. (2008). Design of a solid-state nanopore-based platform for single-molecule spectroscopy. Nanotechnology 19, 165205. 10.1088/0957-4484/19/16/165205 21825639

[B76] Hornblower B. Coombs A. Whitaker R. D. Kolomeisky A. Picone S. J. Meller A.. (2007). Single-molecule analysis of DNA-protein complexes using nanopores. Nat. Meth. 4, 315–317. 10.1038/nmeth1021 17339846

[B77] Huang S. Chang S. He J. Zhang P. Liang F. Tuchband M.. (2010a). Recognition tunneling measurement of the conductance of DNA bases embedded in self-assembled monolayers. J. Phys. Chem. C 114, 20443–20448. 10.1021/jp104792s 21197382 PMC3011824

[B78] Huang S. He J. Chang S. Zhang P. Liang F. Li S.. (2010b). Identifying single bases in a DNA oligomer with electron tunnelling. Nat. Nanotechnol. 5, 868–873. 10.1038/nnano.2010.213 21076404 PMC4121130

[B79] Hyun C. Kaur H. Rollings R. Xiao M. Li J. (2013). Threading immobilized DNA molecules through a solid-state nanopore at >100 μs per base rate. ACS Nano 7, 5892–5900. 10.1021/nn4012434 23758046 PMC3782089

[B80] Ivanov A. P. Freedman K. J. Kim M. J. Albrecht T. Edel J. B. (2014). High precision fabrication and positioning of nanoelectrodes in a nanopore. ACS Nano 8, 1940–1948. 10.1021/nn406586m 24446951

[B81] Ivanov A. P. Instuli E. McGilvery C. M. Baldwin G. McComb D. W. Albrecht T.. (2011). DNA tunneling detector embedded in a nanopore. Nano Lett. 11, 279–285. 10.1021/nl103873a 21133389 PMC3020087

[B82] Jiang Z. Mihovilovic M. Chan J. Stein D. (2010). Fabrication of nanopores with embedded annular electrodes and transverse carbon nanotube electrodes. J. Phys. Condens. Mat. 22, 454114. 10.1088/0953-8984/22/45/454114 21339601

[B83] Karow J. (2009). With new NHGRI grant, Electronic BioSciences focuses on nanopore strand sequencing, in In Sequence/GenomeWeb. Available online at: http://www.genomeweb.com/sequencing/new-nhgri-grant-electronic-bio-sciences-focuses-nanopore-strand-sequencing (Accessed July 8, 2014).

[B84] Karow J. (2010). NobleGen to commercialize BU's optical readout nanopore sequencing tech, in In Sequence/GenomeWeb. Available online at: http://www.genomeweb.com/sequencing/noblegen-commercialize-bus-optical-readout-nanopore-sequencing-tech (Accessed June 16, 2014).

[B85] Karow J. (2011). NobleGen says Optipore tech's fast turnaround, low DNA input, high accuracy will enable clinical apps, in In Sequence/GenomeWeb. Available online at: http://www.genomeweb.com/sequencing/noblegen-says-optipore-techs-fast-turnaround-low-dna-input-high-accuracy-will-en-says-optipore-techs-fast-turnaround-low-dna-input-high-accuracy-will-en (Accessed August 1, 2014).

[B86] Karow J. (2012). Genia licenses Nano-SBS tech from Columbia, NIST; plans 2014 launch of nanopore sequencer, in In Sequence/GenomeWeb. Available online at: http://www.genomeweb.com/sequencing/genia-licenses-nano-sbs-tech-columbia-nist-plans-2014-launch-nanopore-sequencer (Accessed August 1, 2014).

[B87] Karow J. (2014a). First Oxford Nanopore users comment on experience with MinION, start posting data, in In Sequence/GenomeWeb. Available online at: http://www.genomeweb.com/sequencing/first-oxford-nanopore-users-comment-experience-minion-start-posting-data (Accessed June 18, 2014).

[B88] Karow J. (2014b). Japan's Quantum Biosystems shows raw read data from single-molecule nanogap sequencer, in In Sequence/GenomeWeb. Available online at: http://www.genomeweb.com/sequencing/japans-quantum-biosystems-shows-raw-read-data-single-molecule-nanogap-sequencer (Accessed July 12, 2014).

[B89] Karow J. (2014c). Oxford Nanopore presents details on new high-throughput sequencer, improvements to MinIon, in In Sequence/GenomeWeb. Available online at: https://www.genomeweb.com/sequencing/oxford-nanopore-presents-details-new-high-throughput-sequencer-improvements-mini (Accessed November 21, 2014).

[B90] Karow J. (2014d). Oxford Nanopore says 50 kb ‘easily obtained’ from single reads; addresses MinION error types, in In Sequence/GenomeWeb. Available online at: http://www.genomeweb.com/sequencing/oxford-nanopore-says-50-kb-easily-obtained-single-reads-addresses-minion-error-t (Accessed June 16, 2014).

[B91] Karow J. (2014e). Roche broadens sequencing portfolio with Genia acquisition; PacBio partnership continues, in In Sequence/GenomeWeb. Avilable online at: http://www.genomeweb.com/sequencing/roche-broadens-sequencing-portfolio-genia-acquisition-pacbio-partnership-continu (Accessed July 26, 2014).

[B92] Karow J. (2014f). Stratos Genomics working on raising funds to move sequencing-by-expansion technology to market, in In Sequence/GenomeWeb. Available online at: http://www.genomeweb.com/sequencing/stratos-genomics-working-raising-funds-move-sequencing-expansion-technology-mark (Accessed June 16, 2014).

[B93] Kasianowicz J. J. Brandin E. Branton D. Deamer D. W. (1996). Characterization of individual polynucleotide molecules using a membrane channel. Proc. Natl. Acad. Sci. U.S.A. 93, 13770–13773. 10.1073/pnas.93.24.13770 8943010 PMC19421

[B94] Kim J. Maitra R. Pedrotti K. D. Dunbar W. B. (2013a). A patch-clamp ASIC for nanopore-based DNA analysis. IEEE Trans. Biomed. Circuits Syst. 7, 285–295. 10.1109/TBCAS.2012.2200893 23853328

[B95] Kim J. Maitra R. D. Pedrotti K. Dunbar W. B. (2013b). Detecting single-abasic residues within a DNA strand immobilized in a biological nanopore using an integrated CMOS sensor. Sensor Actuat. B Chem. 177, 1075–1082. 10.1016/j.snb.2012.11.027 24496266 PMC3564666

[B96] Kim J. Pedrotti K. Dunbar W. B. (2013c). An area-efficient low-noise CMOS DNA detection sensor for multichannel nanopore applications. Sensor Actuat. B Chem. 176, 1051–1055 10.1016/j.snb.2012.08.075

[B97] Kim J. Pedrotti K. D. Dunbar W. B. (2011). On-chip patch-clamp sensor for solid-state nanopore applications. Electron. Lett. 47, 844–846 10.1049/el.2011.1515

[B98] Kim M. J. Wanunu M. Bell D. C. Meller A. (2006). Rapid fabrication of uniformly sized nanopores and nanopore arrays for parallel DNA analysis. Adv. Mater. 18, 3149–3153 10.1002/adma.200601191

[B99] Korlach J. Turner S. W. (2012). Going beyond five bases in DNA sequencing. Curr. Opin. Chem. Biol. 22, 251–261. 10.1016/j.sbi.2012.04.002 22575758

[B100] Krishnakumar P. Gyarfas B. Song W. Sen S. Zhang P. Krstiæ P.. (2013). Slowing DNA translocation through a nanopore using a functionalized electrode. ACS Nano 7, 10319–10326. 10.1021/nn404743f 24161197 PMC3875158

[B101] Kumar S. Tao C. Chien M. Hellner B. Balijepalli A. Robertson J. W. F.. (2012). PEG-labeled nucleotides and nanopore detection for single molecule DNA sequencing by synthesis. Sci. Rep. 2, 684. 10.1038/srep00684 23002425 PMC3448304

[B102] Kwok H. Briggs K. Tabard-Cossa V. (2014). Nanopore fabrication by controlled dielectric breakdown. PLoS ONE 9:e92880. 10.1371/journal.pone.0092880 24658537 PMC3962464

[B103] Lagerqvist J. Zwolak M. Di Ventra M. (2006). Fast DNA sequencing via transverse electronic transport. Nano Lett. 6, 779–782. 10.1021/nl0601076 16608283 PMC2556950

[B104] Laiwalla F. Klemic K. G. Sigworth F. J. Culurciello E. (2006). An integrated patch-clamp amplifier in silicon-on-sapphire CMOS. IEEE Trans. Circuits Syst. 53, 2364–2370 10.1109/TCSI.2006.884459

[B105] Larkin J. Henley R. Bell D. C. Cohen-Karni T. Rosenstein J. K. Wanunu M. (2013). Slow DNA transport through nanopores in hafnium oxide membranes. ACS Nano 7, 10121–10128. 10.1021/Nn404326f 24083444 PMC4729694

[B106] Laszlo A. H. Derrington I. M. Brinkerhoff H. Langford K. W. Nova I. C. Samson J. M.. (2013). Detection and mapping of 5-methylcytosine and 5-hydroxymethylcytosine with nanopore MspA. Proc. Natl. Acad. Sci. U.S.A. 110, 18904–18909. 10.1073/pnas.1310240110 24167255 PMC3839702

[B107] Laszlo A. H. Derrington I. M. Ross B. C. Brinkerhoff H. Adey A. Nova I. C.. (2014). Decoding long nanopore sequencing reads of natural DNA. Nat. Biotechnol. 32, 829–833. 10.1038/nbt.2950 24964173 PMC4126851

[B108] Lee J. Yang Z. Zhou W. Pennycook S. J. Pantelides S. T. Chisholm M. F. (2014). Stabilization of graphene nanopore. Proc. Natl. Acad. Sci. U.S.A. 111, 7522–7526. 10.1073/pnas.1400767111 24821802 PMC4040544

[B109] Lee J. W. (2007). Nanoelectrode-gated detection of individual molecules with potential for rapid DNA sequencing. Solid State Phenomena 121, 1379–1386. 10.4028/www.scientific.net/SSP.121-123.137923504223

[B110] Lee J. W. Thundat T. G. (2003). DNA and RNA Sequencing by Nanoscale Reading Through Programmable Electrophoresis and Nanoelectrode-Gated Tunneling and Dielectric Detection. US Patent Application Publication No. 2003/0141189, Oak Ridge; Knoxville.

[B111] Leroux A. Destine J. Vanderheyden B. Gracheva M. E. Leburton J. (2010). Spice circuit simulation of the electrical response of a semiconductor membrane to a single-stranded DNA translocating through a nanopore. IEEE Trans. Nanotechnol. 9, 322–329 10.1109/TNANO.2010.2043957

[B112] Li J. Stein D. McMullan C. Branton D. Aziz M. J. Golovchenko J. A. (2001). Ion-beam sculpting at nanometre length scales. Nature 412, 166–169. 10.1038/35084037 11449268

[B113] Li J. Zhang Y. Yang J. Bi K. Ni Z. Li D.. (2013a). Molecular dynamics study of DNA translocation through graphene nanopores. Phys. Rev. E 87, 062707. 10.1103/PhysRevE.87.062707 23848715

[B114] Li W.-W. Gong L. Bayley H. (2013b). Single-molecule detection of 5-hydroxymethylcytosine in DNA through chemical modification and nanopore analysis. Angew. Chem. Int. Ed. 52, 4350–4355. 10.1002/anie.201300413 23559386

[B115] Li W. Bell N. A. Hernandez-Ainsa S. Thacker V. V. Thackray A. M. Bujdoso R.. (2013c). Single protein molecule detection by glass nanopores. ACS Nano 7, 4129–4134. 10.1021/nn4004567 23607870

[B116] Liang F. Li S. Lindsay S. Zhang P. (2012). Synthesis, physicochemical properties, and hydrogen bonding of 4(5)-substituted 1-*H*-imidazole-2-carboxamide, a potential universal reader for DNA sequencing by recognition tunneling. Chem. Euro. J. 18, 5998–6007. 10.1002/chem.201103306 22461259 PMC3367718

[B117] Lieberman K. R. Cherf G. M. Doody M. J. Olasagasti F. Kolodji Y. Akeson M. (2010). Processive replication of single DNA molecules in a nanopore catalyzed by phi29 DNA polymerase. J. Am. Chem. Soc. 132, 17961–17972. 10.1021/ja1087612 21121604 PMC3076064

[B118] Lindsay S. He J. Sankey O. Hapala P. Jelinek P. Zhang P.. (2010). Recognition tunneling. Nanotechnology 21, 262001. 10.1088/0957-4484/21/26/262001 20522930 PMC2891988

[B119] Ling X. S. Bready B. Pertsinidis A. (2006). Hybridization Assisted Nanopore Sequencing. US Patent Application Publication No. 2007/0190542, East Greenwich; Providence.

[B120] Liu H. He J. Tang J. Liu H. Pang P. Cao D.. (2010). Translocation of single-stranded DNA through single-walled carbon nanotubes. Science 327, 64–67. 10.1126/science.1181799 20044570 PMC2801077

[B121] Liu K. Feng J. Kis A. Radenovic A. (2014). Atomically thin molybdenum disulfide nanopores with high sensitivity for DNA translocation. ACS Nano 8, 2504–2511. 10.1021/nn406102h 24547924

[B122] Liu L. Yang C. Zhao K. Li J. Wu H. C. (2013a). Ultrashort single-walled carbon nanotubes in a lipid bilayer as a new nanopore sensor. Nat. Commun. 4, 2989. 10.1038/ncomms3989 24352224 PMC3905707

[B123] Liu S. Lu B. Zhao Q. Li J. Gao T. Chen Y.. (2013b). Boron nitride nanopores: highly sensitive DNA single-molecule detectors. Adv. Mater. 25, 4549–4554. 10.1002/adma.201301336 23775629

[B124] Loman N. Quick J. Calus S. (2014). A p. Aeruginosa serotype-defining single read from our first Oxford Nanopore run, in FigShare. Available online at: http://figshare.com/articles/A_P_aeruginosa_serotype_defining_single_read_from_our_first_Oxford_Nanopore_run/1052996 (Accessed July 30, 2014).

[B125] Lu N. Wang J. Floresca H. C. Kim M. J. (2012). *In situ* studies on the shrinkage and expansion of graphene nanopores under electron beam irradiation at temperatures in the range of 400–1200 °C. Carbon 50, 2961–2965 10.1016/j.carbon.2012.02.078

[B126] Luan B. Martyna G. Stolovitzky G. (2011). Characterizing and controlling the motion of ssDNA in a solid-state nanopore. Biophys. J. 101, 2214–2222. 10.1016/j.bpj.2011.08.038 22067161 PMC3207162

[B127] Luan B. Peng H. Polonsky S. Rossnagel S. Stolovitzky G. Martyna G. (2010). Base-by-base ratcheting of single stranded DNA through a solid-state nanopore. Phys. Rev. Lett. 104, 238103. 10.1103/PhysRevLett.104.238103 20867275 PMC3174011

[B128] Lv W. Liu S. Li X. Wu R. A. (2014). Spatial blockage of ionic current for electrophoretic translocation of DNA through a graphene nanopore. Electrophoresis 35, 1144–1151. 10.1002/elps.201300501 24459097

[B129] Manrao E. A. Derrington I. M. Laszlo A. H. Langford K. W. Hopper M. K. Gillgren N.. (2012). Reading DNA at single-nucleotide resolution with a mutant MspA nanopore and phi29 DNA polymerase. Nat. Biotechnol. 30, 349–353. 10.1038/nbt.2171 22446694 PMC3757088

[B130] Manrao E. A. Derrington I. M. Pavlenok M. Niederweis M. Gundlach J. H. (2011). Nucleotide discrimination with DNA immobilized in the MspA nanopore. PLoS ONE 6:e25723. 10.1371/journal.pone.0025723 21991340 PMC3186796

[B131] Marshall M. M. Ruzicka J. A. Taylor E. W. Hall A. R. (2014). Detecting DNA depurination with solid-state nanopores. PLoS ONE 9:e101632. 10.1371/journal.pone.0101632 24988437 PMC4079296

[B132] Marshall M. M. Yang J. Hall A. R. (2012). Direct and transmission milling of suspended silicon nitride membranes with a focused Helium ion beam. Scanning 34, 101–106. 10.1002/sca.21003 22331671

[B133] Mathé J. Visram H. Viasnoff V. Rabin Y. Meller A. (2004). Nanopore unzipping of individual DNA hairpin molecules. Biophys. J. 87, 3205–3212. 10.1529/biophysj.104.047274 15347593 PMC1304790

[B134] McNally B. Singer A. Yu Z. Sun Y. Weng Z. Meller A. (2010). Optical recognition of converted DNA nucleotides for single-molecule DNA sequencing using nanopore arrays. Nano Lett. 10, 2237–2244. 10.1021/Nl1012147 20459065 PMC2883017

[B135] McNally B. Wanunu M. Meller A. (2008). Electromechanical unzipping of individual DNA molecules using synthetic sub-2 nm pores. Nano Lett. 8, 3418–3422. 10.1021/nl802218f 18759490 PMC2906227

[B136] Meller A. Nivon L. Brandin E. Golovchenko J. Branton D. (2000). Rapid nanopore discrimination between single polynucleotide molecules. Proc. Natl. Acad. Sci. U.S.A. 97, 1079–1084. 10.1073/pnas.97.3.1079 10655487 PMC15527

[B137] Merchant C. A. Healy K. Wanunu M. Ray V. Peterman N. Bartel J.. (2010). DNA translocation through graphene nanopores. Nano Lett. 10, 2915–2921. 10.1021/Nl101046t 20698604

[B138] Meyer J. C. Geim A. K. Katsnelson M. I. Novoselov K. S. Booth T. J. Roth S. (2007). The structure of suspended graphene sheets. Nature 446, 60–63. 10.1038/nature05545 17330039

[B139] Moffitt J. R. Chemla Y. R. Izhaky D. Bustamante C. (2006). Differential detection of dual traps improves the spatial resolution of optical tweezers. Proc. Natl. Acad. Sci. U.S.A. 103, 9006–9011. 10.1073/pnas.0603342103 16751267 PMC1482556

[B140] Muller C. J. Vleeming B. J. Reed M. A. Lamba J. J. S. Hara R. Jones L. (1996). Atomic probes: a search for conduction through a single molecule. Nanotechnology 7, 409 10.1088/0957-4484/7/4/019

[B141] Nam S.-W. Rooks M. J. Kim K.-B. Rossnagel S. M. (2009). Ionic field effect transistors with sub-10 nm multiple nanopores. Nano Lett. 9, 2044–2048. 10.1021/nl900309s 19397298

[B142] Nelson E. M. Li H. Timp G. (2014). Direct, concurrent measurements of the forces and currents affecting DNA in a nanopore with comparable topography. ACS Nano 8, 5484–5493. 10.1021/nn405331t 24840912

[B143] Nelson T. Zhang B. Prezhdo O. V. (2010). Detection of nucleic acids with graphene nanopores: ab initio characterization of a novel sequencing device. Nano Lett. 10, 3237–3242. 10.1021/nl9035934 20722409

[B144] NHGRI (2004). Revolutionary genome sequencing technologies – the $1000 genome, in NIH. Available online at: http://grants.nih.gov/grants/guide/rfa-files/RFA-HG-04-003.html (Accessed July 10, 2014).

[B145] Novoselov K. S. Geim A. K. Morozov S. V. Jiang D. Zhang Y. Dubonos S. V.. (2004). Electric field effect in atomically thin carbon films. Science 306, 666–669. 10.1126/science.1102896 15499015

[B146] Ohshiro T. Matsubara K. Tsutsui M. Furuhashi M. Taniguchi M. Kawai T. (2012). Single-molecule electrical random resequencing of DNA and RNA. Sci. Rep. 2, 501. 10.1038/srep00501 22787559 PMC3392642

[B147] Ohshiro T. Umezawa Y. (2006). Complementary base-pair-facilitated electron tunneling for electrically pinpointing complementary nucleobases. Proc. Natl. Acad. Sci. U.S.A. 103, 10–14. 10.1073/pnas.0506130103 16373509 PMC1324978

[B148] Olasagasti F. Lieberman K. R. Benner S. Cherf G. M. Dahl J. M. Deamer D. W.. (2010). Replication of individual DNA molecules under electronic control using a protein nanopore. Nat. Nanotechnol. 5, 798–806. 10.1038/nnano.2010.177 20871614 PMC3711841

[B149] Ouyang F.-P. Peng S.-L. Zhang H. Weng L.-B. Xu H. (2011). A biosensor based on graphene nanoribbon with nanopores: a first-principles devices-design. Chinese Phys. B 20, 058504 10.1088/1674-1056/20/5/058504

[B150] Pathak B. Löfås H. Prasongkit J. Grigoriev A. Ahuja R. Scheicher R. H. (2012). Double-functionalized nanopore-embedded gold electrodes for rapid DNA sequencing. Appl. Phys. Lett. 100, 023705 10.1063/1.3673335

[B151] Pennisi E. (2012). Search for pore-fection. Science 336, 534–537. 10.1126/science.336.6081.534 22556226

[B152] Polonsky S. Rossnagel S. Stolovitzky G. (2007). Nanopore in metal-dielectric sandwich for DNA position control. Appl. Phys. Lett. 91, 153103 10.1063/1.2798247

[B153] Postma H. W. (2010). Rapid sequencing of individual DNA molecules in graphene nanogaps. Nano Lett. 10, 420–425. 10.1021/nl9029237 20044842

[B154] Prasongkit J. Grigoriev A. Pathak B. Ahuja R. Scheicher R. H. (2011). Transverse conductance of DNA nucleotides in a graphene nanogap from first principles. Nano Lett. 11, 1941–1945. 10.1021/nl200147x 21495701

[B155] Prasongkit J. Grigoriev A. Pathak B. Ahuja R. Scheicher R. H. (2013). Theoretical study of electronic transport through DNA nucleotides in a double-functionalized graphene nanogap. J. Phys. Chem. C 117, 15421–15428 10.1021/jp4048743

[B156] Purnell R. F. Schmidt J. J. (2009). Discrimination of single base substitutions in a DNA strand immobilized in a biological nanopore. Acs Nano 3, 2533–2538. 10.1021/nn900441x 19694456

[B157] Puster M. Rodriguez-Manzo J. A. Balan A. Drndic M. (2013). Toward sensitive graphene nanoribbon-nanopore devices by preventing electron beam-induced damage. ACS Nano 7, 11283–11289. 10.1021/nn405112m 24224888

[B158] Reed M. A. Zhou C. Muller C. J. Burgin T. P. Tour J. M. (1997). Conductance of a molecular junction. Science 278, 252–254 10.1126/science.278.5336.252

[B159] Reiner J. E. Balijepalli A. Robertson J. W. F. Drown B. S. Burden D. L. Kasianowicz J. J. (2012). The effects of diffusion on an exonuclease/nanopore-based DNA sequencing engine. J. Chem. Phys. 137, 214903. 10.1063/1.4766363 23231259 PMC4108639

[B160] Rodriguez-Manzo J. A. Banhart F. (2009). Creation of individual vacancies in carbon nanotubes by using an electron beam of 1 Å diameter. Nano Lett. 9, 2285–2289. 10.1021/nl900463u 19413339

[B161] Rosenstein J. K. Shepard K. L. (2013). Temporal resolution of nanopore sensor recordings, in Engineering in Medicine and Biology Society (EMBC), 35th Annual International Conference of the IEEE (Osaka). 10.1109/EMBC.2013.6610449 24110636

[B162] Rosenstein J. K. Wanunu M. Merchant C. A. Drndic M. Shepard K. L. (2012). Integrated nanopore sensing platform with sub-microsecond temporal resolution. Nat. Meth. 9, 487–492. 10.1038/nmeth.1932 22426489 PMC3648419

[B163] Russo C. J. Golovchenko J. A. (2012). Atom-by-atom nucleation and growth of graphene nanopores. Proc. Natl. Acad. Sci. U.S.A. 109, 5953–5957. 10.1073/pnas.1119827109 22492975 PMC3340994

[B164] Sadeghi H. Algaragholy L. Pope T. Bailey S. Visontai D. Manrique D.. (2014). Graphene sculpturene nanopores for DNA nucleobase sensing. J. Phys. Chem. B 118, 6908–6914. 10.1021/jp5034917 24849015

[B165] Sadki E. S. Garaj S. Vlassarev D. Golovchenko J. A. Branton D. (2011). Embedding a carbon nanotube across the diameter of a solid state nanopore. J. Vac. Sci. Technol. B 29, 053001 10.1116/1.3628602

[B166] Saha K. K. Drndić M. Nikolić B. K. (2012). DNA base-specific modulation of microampere transverse edge currents through a metallic graphene nanoribbon with a nanopore. Nano Lett. 12, 50–55. 10.1021/nl202870y 22141739 PMC3272331

[B167] Sauer-Budge A. F. Nyamwanda J. A. Lubensky D. K. Branton D. (2003). Unzipping kinetics of double-stranded DNA in a nanopore. Phys. Rev. Lett. 90, 238101. 10.1103/PhysRevLett.90.238101 12857290

[B168] Saunders C. J. Miller N. A. Soden S. E. Dinwiddie D. L. Noll A. Alnadi N. A.. (2012). Rapid whole-genome sequencing for genetic disease diagnosis in neonatal intensive care units. Sci. Transl. Med. 4, 154ra135. 10.1126/scitranslmed.3004041 23035047 PMC4283791

[B169] Sawafta F. Clancy B. Carlsen A. T. Huber M. Hall A. R. (2014). Solid-state nanopores and nanopore arrays optimized for optical detection. Nanoscale 6, 6991–6996. 10.1039/c4nr00305e 24838772

[B170] Schadt E. E. Turner S. Kasarskis A. (2010). A window into third-generation sequencing. Hum. Mol. Genet. 19, R227–R240. 10.1093/hmg/ddq416 20858600

[B171] Schibel A. E. P. An N. Jin Q. Fleming A. M. Burrows C. J. White H. S. (2010). Nanopore detection of 8-oxo-7,8-dihydro-2′-deoxyguanosine in immobilized single-stranded DNA via adduct formation to the DNA damage site. J. Am. Chem. Soc. 132, 17992–17995. 10.1021/ja109501x 21138270 PMC3021242

[B172] Schneider G. F. Dekker C. (2012). DNA sequencing with nanopores. Nat. Biotechnol. 30, 326–328. 10.1038/nbt.2181 22491281

[B173] Schneider G. F. Kowalczyk S. W. Calado V. E. Pandraud G. Zandbergen H. W. Vandersypen L. M.. (2010). DNA translocation through graphene nanopores. Nano Lett. 10, 3163–3167. 10.1021/Nl102069z 20608744

[B174] Schneider G. F. Xu Q. Hage S. Luik S. Spoor J. N. H. Malladi S.. (2013). Tailoring the hydrophobicity of graphene for its use as nanopores for DNA translocation. Nat. Commun. 4, 2619. 10.1038/ncomms3619 24126320

[B175] Schreiber J. Wescoe Z. L. Abu-Shumays R. Vivian J. T. Baatar B. Karplus K.. (2013). Error rates for nanopore discrimination among cytosine, methylcytosine, and hydroxymethylcytosine along individual DNA strands. Proc. Natl. Acad. Sci. U.S.A. 110, 18910–18915. 10.1073/pnas.1310615110 24167260 PMC3839712

[B176] Seo T. S. Bai X. Kim D. H. Meng Q. Shi S. Ruparel H.. (2005). Four-color DNA sequencing by synthesis on a chip using photocleavable fluorescent nucleotides. Proc. Natl. Acad. Sci. U.S.A. 102, 5926–5931. 10.1073/pnas.0501965102 15829588 PMC1087949

[B177] Sha J. Hasan T. Milana S. Bertulli C. Bell N. A. Privitera G.. (2013). Nanotubes complexed with DNA and proteins for resistive-pulse sensing. ACS Nano 7, 8857–8869. 10.1021/nn403323k 24066614

[B178] Shendure J. Porreca G. J. Reppas N. B. Lin X. McCutcheon J. P. Rosenbaum A. M.. (2005). Accurate multiplex polony sequencing of an evolved bacterial genome. Science 309, 1728–1732. 10.1126/science.1117389 16081699

[B179] Sigalov G. Comer J. Timp G. Aksimentiev A. (2008). Detection of DNA sequences using an alternating electric field in a nanopore capacitor. Nano Lett. 8, 56–63. 10.1021/nl071890k 18069865 PMC2588427

[B180] Sint K. Wang B. KráaL P. (2008). Selective ion passage through functionalized graphene nanopores. J. Am. Chem. Soc. 130, 16448–16449. 10.1021/ja804409f 19554715

[B181] Siwy Z. Fuliñski A. (2002). Fabrication of a synthetic nanopore ion pump. Phys. Rev. Lett. 89, 198103. 10.1103/PhysRevLett.89.198103 12443155

[B182] Siwy Z. S. Davenport M. (2010). Nanopores: graphene opens up to DNA. Nat. Nanotechnol. 5, 697–698. 10.1038/nnano.2010.198 20924388

[B183] Smith S. B. Cui Y. Bustamante C. (1996). Overstretching B-DNA: the elastic response of individual double-stranded and single-stranded DNA molecules. Science 271, 795–799. 10.1126/science.271.5250.795 8628994

[B184] Song B. Schneider G. F. Xu Q. Pandraud G. Dekker C. Zandbergen H. (2011). Atomic-scale electron-beam sculpting of near-defect-free graphene nanostructures. Nano Lett. 11, 2247–2250. 10.1021/Nl200369r 21604710

[B185] Song L. Hobaugh M. R. Shustak C. Cheley S. Bayley H. Gouaux J. E. (1996). Structure of staphylococcal α-hemolysin, a heptameric transmembrane pore. Science 274, 1859–1865. 10.1126/science.274.5294.1859 8943190

[B186] Soni G. V. Meller A. (2007). Progress toward ultrafast DNA sequencing using solid-state nanopores. Clin. Chem. 53, 1996–2001. 10.1373/clinchem.2007.091231 17890440

[B187] Spencer G. (2010). NHGRI seeks next generation of sequencing technologies, in NHGRI. Available online at: http://www.genome.gov/12513210 (Accessed July 8, 2014).

[B188] Spinney P. S. Collins S. D. Howitt D. G. Smith R. L. (2012). Fabrication and characterization of a solid-state nanopore with self-aligned carbon nanoelectrodes for molecular detection. Nanotechnology 23:135501. 10.1088/0957-4484/23/13/135501 22421078

[B189] Spinney P. S. Collins S. D. Smith R. L. Howitt D. G. (2009). Electrical characterization of a carbon nanoelectrode instrumented nanopore sensor, in Sensors, 8th Conference of IEEE (Christchurch).

[B190] Stoddart D. Heron A. J. Klingelhoefer J. Mikhailova E. Maglia G. Bayley H. (2010a). Nucleobase recognition in ssDNA at the central constriction of the α-hemolysin pore. Nano Lett. 10, 3633–3637. 10.1021/nl101955a 20704324 PMC2935931

[B191] Stoddart D. Heron A. J. Mikhailova E. Maglia G. Bayley H. (2009). Single-nucleotide discrimination in immobilized DNA oligonucleotides with a biological nanopore. Proc. Natl. Acad. Sci. U.S.A. 106, 7702–7707. 10.1073/pnas.0901054106 19380741 PMC2683137

[B192] Stoddart D. Maglia G. Mikhailova E. Heron A. J. Bayley H. (2010b). Multiple base-recognition sites in a biological nanopore: two heads are better than one. Angew. Chem. Int. Ed. 49, 556–559. 10.1002/anie.200905483 20014084 PMC3128935

[B193] Storm A. J. Chen J. H. Ling X. S. Zandbergen H. W. Dekker C. (2003). Fabrication of solid-state nanopores with single-nanometre precision. Nat. Mater. 2, 537–540. 10.1038/nmat941 12858166

[B194] Suk M. E. Aluru N. R. (2014). Ion transport in sub-5-nm graphene nanopores. J. Chem. Phys. 140, 084707. 10.1063/1.4866643 24588191

[B195] Sutanto J. Smith R. Collins S. (2010). Fabrication of nano-gap electrodes and nano-wires by using electrochemical and chemical etching technique for a nano-pore DNA/RNA sequencer, in Medical Device Materials V: Proceedings of the Materials & Processes for Medical Devices Conference (Minneapolis, MN).

[B196] Tanaka H. Kawai T. (2009). Partial sequencing of a single DNA molecule with a scanning tunnelling microscope. Nat. Nanotechnol. 4, 518–522. 10.1038/nnano.2009.155 19662015

[B197] Tian K. He Z. Wang Y. Chen S.-J. Gu L.-Q. (2013). Designing a polycationic probe for simultaneous enrichment and detection of micrornas in a nanopore. ACS Nano 7, 3962–3969. 10.1021/nn305789z 23550815 PMC3675772

[B198] Toner B. (2012). Prepping beta version of nanopore sequencer, Genia touts semiconductor platform as differentiator, in In Sequence/GenomeWeb. Avilable online at: http://www.genomeweb.com/sequencing/prepping-beta-version-nanopore-sequencer-genia-touts-semiconductor-platform-diff (Accessed July 8, 2014).

[B199] Topol E. J. (2014). Individualized medicine from prewomb to tomb. Cell 157, 241–253. 10.1016/j.cell.2014.02.012 24679539 PMC3995127

[B200] Torre R. Larkin J. Singer A. Meller A. (2012). Fabrication and characterization of solid-state nanopore arrays for high-throughput DNA sequencing. Nanotechnology 23:385308. 10.1088/0957-4484/23/38/385308 22948520 PMC3557807

[B201] Traversi F. Raillon C. Benameur S. M. Liu K. Khlybov S. Tosun M.. (2013). Detecting the translocation of DNA through a nanopore using graphene nanoribbons. Nat. Nanotechnol. 8, 939–945. 10.1038/Nnano.2013.240 24240429

[B202] Treffer R. Lin X. Bailo E. Deckert-Gaudig T. Deckert V. (2011). Distinction of nucleobases – a tip-enhanced Raman approach. Beilstein J. Nanotechnol. 2, 628–637. 10.3762/bjnano.2.66 22003468 PMC3190632

[B203] Tsutsui M. Matsubara K. Ohshiro T. Furuhashi M. Taniguchi M. Kawai T. (2011a). Electrical detection of single methylcytosines in a DNA oligomer. J. Am. Chem. Soc. 133, 9124–9128. 10.1021/ja203839e 21561093

[B204] Tsutsui M. Rahong S. Iizumi Y. Okazaki T. Taniguchi M. Kawai T. (2011b). Single-molecule sensing electrode embedded in-plane nanopore. Sci. Rep. 1, 46. 10.1038/srep00046 22355565 PMC3216533

[B205] Tsutsui M. Taniguchi M. Kawai T. (2008). Fabrication of 0.5 nm electrode gaps using self-breaking technique. Appl. Phys. Lett. 93, 163115 10.1063/1.3006063

[B206] Tsutsui M. Taniguchi M. Kawai T. (2009). Transverse field effects on DNA-sized particle dynamics. Nano Lett. 9, 1659–1662. 10.1021/nl900177q 19256477

[B207] Tsutsui M. Taniguchi M. Yokota K. Kawai T. (2010). Identifying single nucleotides by tunnelling current. Nat. Nanotechnol. 5, 286–290. 10.1038/nnano.2010.42 20305643

[B208] Venkatesan B. M. Bashir R. (2011). Nanopore sensors for nucleic acid analysis. Nat. Nanotechnol. 6, 615–624. 10.1038/nnano.2011.129 21926981

[B209] Venta K. Shemer G. Puster M. Rodriguez-Manzo J. A. Balan A. Rosenstein J. K.. (2013). Differentiation of short, single-stranded DNA homopolymers in solid-state nanopores. ACS Nano 7, 4629–4636. 10.1021/nn4014388 23621759 PMC3724363

[B210] Vogelstein B. Papadopoulos N. Velculescu V. E. Zhou S. Diaz L. A. Kinzler K. W. (2013). Cancer genome landscapes. Science 339, 1546–1558. 10.1126/science.1235122 23539594 PMC3749880

[B211] Wallace E. V. B. Stoddart D. Heron A. J. Mikhailova E. Maglia G. Donohoe T. J.. (2010). Identification of epigenetic DNA modifications with a protein nanopore. Chem. Comm. 46, 8195–8197. 10.1039/C0CC02864A 20927439 PMC3147113

[B212] Wang Z. (2010). A Method for Sequencing of Single-Molecule Nucleic Acids Using Exonulease-Nanopre. PRC Patent No. CN1932039, Shanghai.

[B213] Wang Z. Huang S. (2010). A 2D-Tuneable Nanopore Fabrication Method by Carbon Nanotubes. PRC Patent No. CN1994864, Shanghai; Wenzhou.

[B214] Wanunu M. (2012). Nanopores: a journey towards DNA sequencing. Phys. Life Rev. 9, 125–158 10.1016/j.plrev.2012.05.010 22658507 PMC3780799

[B215] Wanunu M. Cohen-Karni D. Johnson R. R. Fields L. Benner J. Peterman N.. (2010a). Discrimination of methylcytosine from hydroxymethylcytosine in DNA molecules. J. Am. Chem. Soc. 133, 486–492. 10.1021/ja107836t 21155562 PMC3081508

[B216] Wanunu M. Dadosh T. Ray V. Jin J. McReynolds L. Drndić M. (2010b). Rapid electronic detection of probe-specific microRNAs using thin nanopore sensors. Nat. Nanotechnol. 5, 807–814. 10.1038/Nnano.2010.202 20972437

[B217] Weerakoon P. Culurciello E. Klemic K. G. Sigworth F. J. (2009). An integrated patch-clamp potentiostat with electrode compensation. IEEE Trans Biomed Circuits Syst 3, 117–125. 10.1109/TBCAS.2008.2005419 23853203

[B218] Weerakoon P. Culurciello E. Yang Y. Santos-Sacchi J. Kindlmann P. J. Sigworth F. J. (2010). Patch-clamp amplifiers on a chip. J. Neurosci. Meth. 192, 187–192. 10.1016/j.jneumeth.2010.06.030 20637803 PMC2978236

[B219] Wells D. B. Belkin M. Comer J. Aksimentiev A. (2012). Assessing graphene nanopores for sequencing DNA. Nano Lett 12, 4117–4123. 10.1021/nl301655d 22780094 PMC3434709

[B220] Wilson N. A. Abu-Shumays R. Gyarfas B. Wang H. Lieberman K. R. Akeson M.. (2009). Electronic control of DNA polymerase binding and unbinding to single DNA molecules. ACS Nano 3, 995–1003. 10.1021/nn9000897 19338283 PMC2708927

[B221] Wu J. Zhang S. Meng Q. Cao H. Li Z. Li X.. (2007). 3′-O-modified nucleotides as reversible terminators for pyrosequencing. Proc. Natl. Acad. Sci. U.S.A. 104, 16462–16467. 10.1073/pnas.0707495104 17923668 PMC2034218

[B222] Xie P. Xiong Q. Fang Y. Qing Q. Lieber C. M. (2012). Local electrical potential detection of DNA by nanowire-nanopore sensors. Nat. Nanotechnol. 7, 119–125. 10.1038/nnano.2011.217 22157724 PMC3273648

[B223] Xu M. Endres R. G. Arakawa Y. (2007). The electronic properties of DNA bases. Small 3, 1539–1543. 10.1002/smll.200600732 17786897

[B224] Xu M. Liang T. Shi M. Chen H. (2013a). Graphene-like two-dimensional materials. Chem. Rev. 113, 3766–3798. 10.1021/cr300263a 23286380

[B225] Xu M. S. Endres R. G. Tsukamoto S. Kitamura M. Ishida S. Arakawa Y. (2005). Conformation and local environment dependent conductance of DNA molecules. Small 1, 1168–1172. 10.1002/smll.200500216 17193411

[B226] Xu Q. Wu M.-Y. Schneider G. F. Houben L. Malladi S. K. Dekker C.. (2013b). Controllable atomic scale patterning of freestanding monolayer graphene at elevated temperature. ACS Nano 7, 1566–1572. 10.1021/nn3053582 23343745

[B227] Xu T. Yin K. Xie X. He L. Wang B. Sun L. (2012). Size-dependent evolution of graphene nanopores under thermal excitation. Small 8, 3422–3426. 10.1002/smll.201200979 22903811

[B228] Yanagi I. Akahori R. Hatano T. Takeda K.-I. (2014). Fabricating nanopores with diameters of sub-1 nm to 3 nm using multilevel pulse-voltage injection. Sci. Rep. 4:5000. 10.1038/srep05000 24847795 PMC4028839

[B229] Yokota K. Tsutsui M. Taniguchi M. (2014). Electrode-embedded nanopores for label-free single-molecule sequencing by electric currents. RSC Adv. 4, 15886–15899. 10.1039/C4RA00933A22355565

[B230] Zhang Z. Shen J. Wang H. Wang Q. Zhang J. Liang L. (2014). Effects of graphene nanopore geometry on DNA sequencing. J. Phys. Chem. Lett 5, 1602–1607 10.1021/jz500498c 26270103

[B231] Zhao X. Liu Y. Inoue S. Suzuki T. Jones R. O. Ando Y. (2004). Smallest carbon nanotube is 3 Å in diameter. Phys. Rev. Lett. 92, 125502. 10.1103/PhysRevLett.92.125502 15089683

[B232] Zhao Y. Ashcroft B. Zhang P. Liu H. Sen S. Song W.. (2014). Single-molecule spectroscopy of amino acids and peptides by recognition tunnelling. Nat. Nanotechnol. 9, 466–473. 10.1038/nnano.2014.54 24705512 PMC4047173

[B233] Zhou Z. Hu Y. Wang H. Xu Z. Wang W. Bai X.. (2013). DNA translocation through hydrophilic nanopore in hexagonal boron nitride. Sci. Rep. 3, 3287. 10.1038/srep03287 24256703 PMC3836030

[B234] Zhu Z. Tumati R. Collins S. Smith R. Kotecki D. E. (2006). A low-noise low-offset op amp in 0.35 μm CMOS process, in Electronics, Circuits and Systems, 13th IEEE International Conference (Nice).

[B235] Zwolak M. Di Ventra M. (2005). Electronic signature of DNA nucleotides via transverse transport. Nano Lett. 5, 421–424. 10.1021/nl048289w 15755087

